# Stabilizing Carbon Nitride Photoanodes for Unassisted Alcohol Reforming Coupled to CO_2_ Reduction under Concentrated Sunlight

**DOI:** 10.1021/jacs.5c20624

**Published:** 2026-03-17

**Authors:** Carolina Pulignani, Ariffin Bin Mohamad Annuar, Samuel J. Cobb, Motiar Rahaman, Yongpeng Liu, Chen Han, Andrea Rogolino, Subhajit Bhattacharjee, Erwin Reisner

**Affiliations:** Yusuf Hamied Department of Chemistry, 2152 University of Cambridge, Lensfield Road, Cambridge CB2 1EW, U.K.

## Abstract

Carbon nitride is a scalable and low-cost organic semiconductor with promise for use in photoelectrochemical (PEC) devices, but its poor stability limits its practical use. This study establishes the importance of light intensity and applied electrochemical potential on the stability of cyanamide-functionalized carbon nitride photoanodes used for waste-derived alcohol oxidation. Operating under concentrated sunlight (3 suns) and low bias (<0.2 V vs reversible hydrogen electrode, RHE) enabled over 20 h of continuous operation. *In situ* spectroscopy and impedance analysis linked carbon nitride degradation at higher potential (>0.4 V vs RHE) to irreversible morphological changes and self-oxidation, exacerbated by conductivity loss. These insights enabled the stabilization of carbon nitride photoelectrodes and paved the way for the development of a standalone PEC device with an integrated thermoelectric unit to convert heat into an additional internal bias-voltage, where carbon nitride efficiently drives alcohol oxidation coupled to CO_2_-to-CO conversion for over 70 h. This work sets a benchmark for carbon nitride stability and PEC deployment, and highlights the need to revisit PEC testing protocols to unlock the full potential of emerging semiconductors.

## Graphical abstract

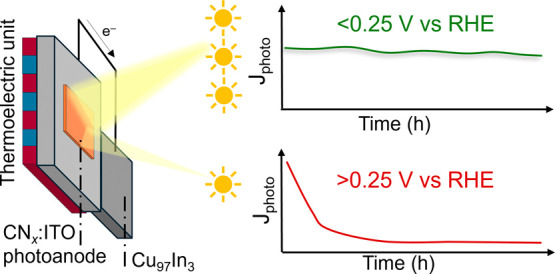

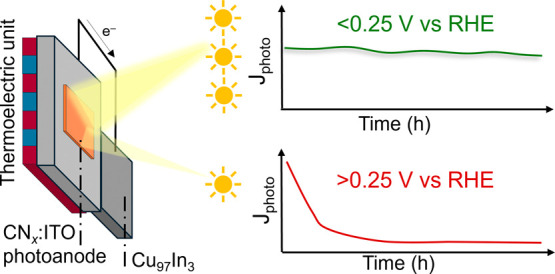

## Introduction

The urgent need for clean energy solutions has driven significant research into sustainable photoelectrochemical (PEC) technologies, a promising strategy to directly capture and utilize sunlight to drive chemical transformations in a single device.^1–4^ However, achieving efficient and long-lasting photoelectrodes remains a challenge, particularly when using nontoxic, inexpensive, and scalable nonoxide semiconductors.^5,6^

Carbon nitride (CN_*x*_) holds particular promise as semiconductor for PEC applications, especially for water and organic substrate oxidation.^7–9^ CN_*x*_ materials combine high chemical and thermal stability, minimal toxicity, and tunable visible light absorption with easy and low-cost synthesis from earth-abundant precursors.^10^ The best performing CN_*x*_ photoanodes now reach photocurrents of 0.9 mA cm^–2^ (at 1.23 V vs reversible hydrogen electrode, RHE) for water oxidation (WO),^11,12^ and up to 1.4 mA cm^–2^ (at 1.23 V vs RHE) were reported with sacrificial electron donors (e.g., triethanolamine, 4-methylbenzyl alcohol (4-MBA), methanol),^13,14^ or the valorization of organics (e.g., glycerol and ethylene glycol, PET monomer).^15^

However, the major limitation of CN_*x*_ photoanodes remains, which is their poor operational stability.^16^ Although strategies such as cocatalyst addition,^17–19^ doping,^20,21^ or heterojunction formation with an additional semiconductor can extend stability,^21–23^ most examples of PEC devices employing solely CN_*x*_ as semiconductor operate for less than 3 h,^11,24–27^ or lose 40% of activity within 10 h.^13,28–30^ The instability of CN_*x*_ photoanodes has been attributed to (i) physical detaching of CN_*x*_ from the support due to poor film adhesion and inhomogeneity;^31^ (ii) self-oxidation, resulting in C–N bond breaking and reduced oxidation ability;^32^ (iii) low intrinsic conductivity, leading to electron accumulation and internal resistance buildup;^28,33^ or (iv) irreversible side chemical reactions involving long-lived radical species within the ionic CN_*x*_ polymeric structure, poisoning the photoelectrode surface.^34,35^

In this work, we systematically investigated the stability of CN_*x*_ photoanodes made of ionic cyanamide-functionalized carbon nitride (a class of poly­(heptazine imide) carbon nitrides, PHIs), focusing on the effect of light intensity and applied electrochemical potential on their long-term performance during waste-derived alcohol oxidation. This overlooked approach enabled us to identify conditions, i.e., a potential range between 0 and 0.2 V vs RHE and light intensity between 200 and 300 mW cm^–2^ (2–3 suns), which stabilize the photoanodes at conditions relevant for use in PEC devices. Thus, we could bypass CN_*x*_ photoanode instability by carefully selecting operationally relevant conditions that were previously not considered by standard experimental protocols, paving the way for stable CN_*x*_ photoelectrochemistry. *In situ* infrared and voltage-dependent PEC impedance spectroscopy provided qualitative insights into photo- and electrochemical processes leading to long-term stability. Establishing a stable glycerol-oxidizing CN_*x*_ photoanode allowed coupling to CO_2_ reduction to CO by a cathode made from a copper–indium electrocatalyst (Cu_97_In_3_). This CN_*x*_-based PEC device can operate without external applied voltage for more than 70 h by integrating a thermoelectric generator (TEG). The TEG enables advanced light management by utilizing IR in the form of waste heat from the concentrated solar irradiation to provide the additional internal bias required for CO_2_ reduction over H_2_ evolution,^36^ while UV and visible light photoexcite the CN_*x*_ photoanode (Figure 1).

**Figure 1:**
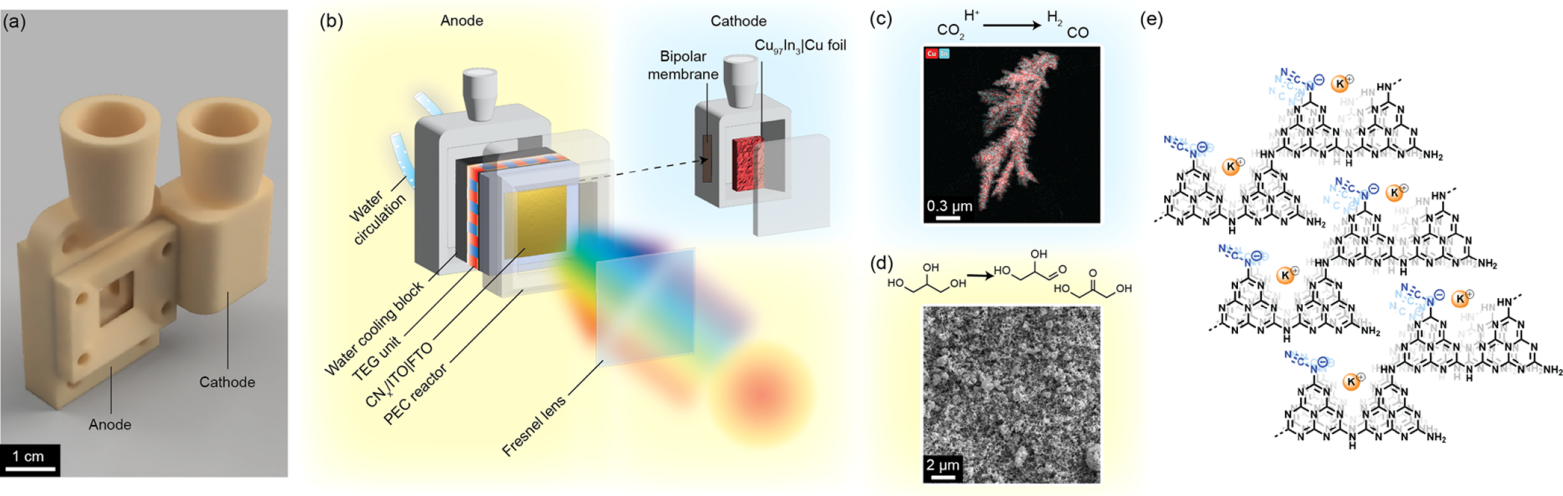
Overview of TEG-PEC cell design. (a) Front view of the CAD rendering of the assembled TEG-PEC cell. (b) Schematic representation of the integration of the stabilized CN_*x*_/ITO photoanode into a customized TEG-PEC cell for unbiased operation under concentrated solar light. (c) EDX mapping of the Cu_97_In_3_ dark cathode for the reduction of CO_2_ into CO, and protons to H_2_. Colors highlight the uniform distribution of copper (red) and indium (light blue) elemental distribution. (d) Top-view SEM images of CN_*x*_/ITO photoanode (1 cm^2^ active area used in TEG-PEC device) for the valorization of glycerol into glyceraldehyde and dihydroxyacetone. (e) Chemical structure of cyanamide-functionalized carbon nitride (CN_*x*_).

## Results and Discussion

### Analysis of Photoanode Performance

Cyanamide-functionalized ionic carbon nitride (in this work simplified as CN_*x*_),^37^ synthesized by postfunctionalization of melamine-derived graphitic carbon nitride with potassium thiocyanate,^38,39^ was mixed in equal weight with a conductive binder (commercial indium tin oxide nanoparticles, ITO) and drop-cast on FTO-coated glass. This carbon nitride photoanode was previously optimized to enhance charge separation and minimize charge-extraction resistance, yielding high photocurrent densities.^13^ This synthetic protocol was selected as the photoanode architecture has been systematically validated to ensure homogeneous film coverage, reproducible thicknesses, and consistent PEC performance across multiple independently prepared samples. Glycerol was selected as the electron donor because the selected CN_*x*_ exhibits excellent activity for alcohol oxidation,^13,40^ and the oxidative valorization of glycerol, a waste product from biodiesel production, provides an attractive means for sustainable chemistry.^41,42^

Despite the importance of investigating the effects of light intensity and applied potential to obtain insights into the fundamental processes governing PEC performance,^43^ their systematic analysis has not yet been reported for CN_*x*_ photoanodes. Previous studies are therefore limited to optimizing photocurrents, and chronoamperometry (CA) tests are routinely performed under 1 sun and potentials >1 V vs RHE (V_RHE_), even with organic oxidation substrates (i.e., triethanolamine, 4-MBA, glycerol, and methanol).^44^ We note that those potentials are not meaningful for operation in a two-electrode PEC device, at least when coupling to a fuel-forming reaction such as proton or CO_2_ reduction (see below).

Thus, to understand performance bottlenecks and to identify optimal operating conditions, the long-term stability of our CN_*x*_/ITO photoanodes was studied under different light intensities and applied potentials. PEC tests were performed in a three-electrode configuration, with a Pt mesh as counter and a Ag/AgCl (sat. KCl) as reference electrode, in 5 v/v % glycerol (0.68 M) in aqueous Na_2_SO_4_ (0.1 M, pH 7) solution. The light intensity (Air Mass 1.5 Global) was increased by concentrating the radiation with a commercial Fresnel lens (details in Experimental Section, Supporting Information). All PEC experiments were carried out in a gastight cell, purging the electrolyte with N_2_ (with 2% CH_4_) for at least 20 min prior to measurement.

First, linear sweep voltammetry (LSV) studies of the CN_*x*_/ITO photoanodes were performed under 1 and 3 suns (Figure 2a). Increasing the light intensity from 1 (0.9 mA cm^–2^ at 1.2 V_RHE_) to 3 suns (1.15 mA cm^–2^) did not result in a proportional photocurrent increase. This behavior may be consistent with previous studies on this photoanode, which identified the conductivity of the mechanical and electrical binder, viz. ITO, as the performance bottleneck, limiting the photocurrent enhancement despite higher light intensity.^13^

**Figure 2:**
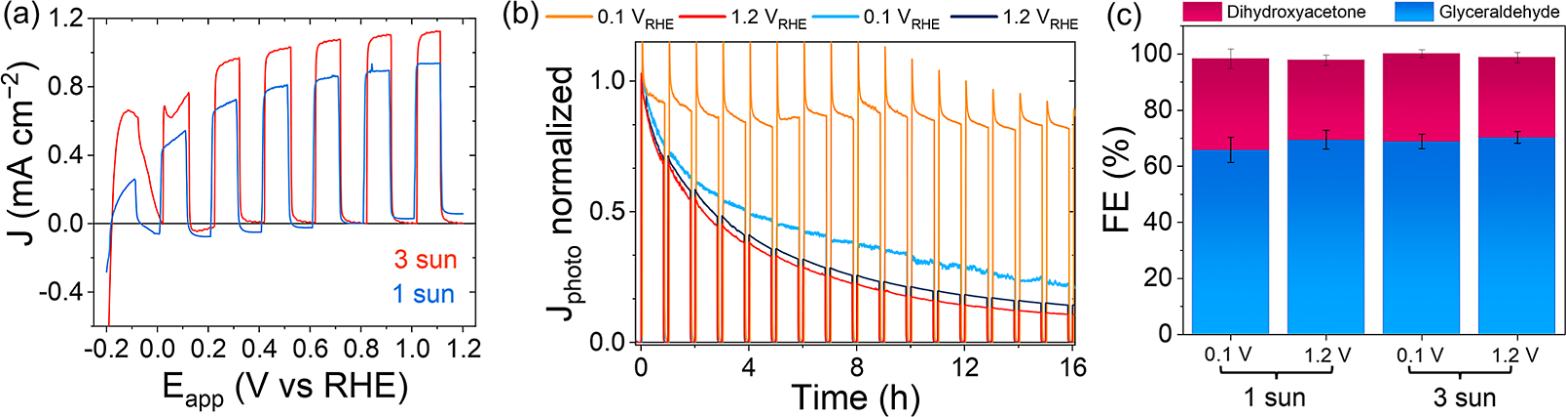
PEC analysis of CN*_x_*/ITO photoanode as a function of light intensity and applied potential. (a) LSV scans of a CN_*x*_/ITO working electrode in a 3-electrode setup under 1 sun (blue trace) and 3 suns (red trace), scan rate 10 mV s^–1^. (b) Normalized chopped CA traces with 0.1 and 1.2 V vs RHE applied potential in a 3-electrode setup under 1 (light and dark blue traces) and 3 suns (orange and red traces). (c) FE analysis of glycerol oxidation products under different light concentrations and applied potentials. Conditions (a–c): 0.25 and 0.5 cm^–2^ electrode area (front illumination) used interchangeably throughout, Pt mesh as counter electrode, Ag/AgCl (KCl saturated) as reference electrode, 5 v/v % glycerol (0.68 M) in Na_2_SO_4_ aqueous solution (0.1 M, pH 7), chopped simulated solar light (100 or 300 mW cm^–2^) with 10 s on/10 s off for the LSV scans (10 mV s^–1^) and 50 min on/10 min off for the CA measurements. Example traces are given in (a,b). The temperature of the electrolyte solution reached 40 °C after 16 h under concentrated solar light (3 suns) and 28 °C under 1 sun. The pH did not change after any CA tests. Measurements were performed in triplicate.

The stability of the photoanodes was then evaluated by applying either 0.1 V_RHE_ or 1.2 V_RHE_, where the latter is the literature standard potential to study CN_*x*_ photoanodes. We note that 1.2 V_RHE_ is approximately the standard potential (*E* ^0^) for water oxidation and, consequently, a far too positive potential for photoanode operation in a bias-free two-electrode PEC cell for solar energy storage involving O_2_ evolution and, even more so, for organic oxidations.

The normalized photocurrent responses allow for direct comparison between the four tested conditions (Figure 2b). Under 1 sun, rapid photocurrent decay occurred at both potentials, with >70% current loss over 15 h, consistent with most CN_*x*_ photoanodes reported so far. Unexpectedly, the photoanode displayed unprecedented long-term stability at 3 suns and 0.1 V_RHE_, maintaining 0.32 ± 0.01 mA cm^–2^ for 20 h followed by a decrease to 0.20 ± 0.02 mA cm^–2^ after 26 h (Figure S1). A comparable stability trend was also observed under 2 suns at 0.1 V_RHE_ (Figure S2 with extended discussion). However, at 1.2 V_RHE_ and 3 suns, the photocurrent declined rapidly from 1.28 to 0.13 mA cm^–2^, losing >80% after 20 h (Figure 2b). Despite differences in photocurrent decay, the total faradaic efficiency (FE, 95 ± 3%) remained constant across all conditions, with a 2:1 product ratio between glyceraldehyde and dihydroxyacetone (Figure 2c).

Inductively coupled plasma optical emission spectroscopy (ICP-OES) analysis showed increased indium (from ITO) leaching at 1.2 V_RHE_, while minimal loss occurred at 0.1 V_RHE_ under 3 suns (Figure S3). CO evolution was also monitored during the 3-sun CA tests, and traces (5–30 nmol) were observed only at high potentials after 20 h (Figure S4).

Temperature-controlled studies (25–70 °C) confirmed that heat generated by concentrated solar light is not the primary driver of improved stability at low applied potentials. Namely, the characteristic exponential current decay was observed at 1 sun across all tested temperatures with both glycerol and 4-MBA (Figures S5 and S6 with extended discussion). These control experiments indicate that the oxidation substrate (electron donor) itself does not govern the enhanced long-term stability. In addition, together with the nonlinear photocurrent response at increasing light intensities (Figure S1), these results suggest that the improved stability observed under concentrated illumination cannot be attributed solely to enhanced reaction kinetics but to the simultaneous optimization of light and applied potential.

CA at increasing potentials (0.1–0.8 V_RHE_) under 2 and 3 suns revealed good photoelectrode stability up to a threshold potential of ∼0.25 V_RHE_ (Figure S7 and extended discussion). Control experiments in the dark confirmed that the deactivation is mainly an electrochemical process, promoted further by light (Figures S8 and S9). PEC measurements performed at lower light intensities (0.5 sun) further support the stabilizing role of concentrated illumination, as the characteristic rapid photocurrent decay was observed at both low and high applied potentials (Figure S10). Moreover, the nonrecoverability of the initial photocurrent after applying high potentials suggests irreversible changes in the CN_*x*_ (electro)­chemical structure (Figure S11).

### Spectroscopic Study

To elucidate how the external applied potential affects CN_*x*_/ITO photoanode stability, *in situ* synchrotron radiation-Fourier transform infrared (SR-FTIR) spectroscopy measurements were conducted at 0.2 and 1.2 V_RHE_.^45^ The SR-FTIR setup only allowed for testing under 1 sun front illumination in a customized PEC cell (further details and discussion are available in Supporting Information). A lower glycerol concentration (50 mM) was employed to minimize possible interference in the IR spectra related to the two main products, whose absorption peaks fall between 1800 and 500 cm^–1^.^46–48^

The IR absorption spectra were collected every 4 min during 1 h CA at 0.2 or 1.2 V_RHE_ (Figure S12). To allow for a direct comparison of the relative change in vibrational modes over time, the time-zero spectrum of each testing condition was subtracted from each *t* > 4 min spectrum (Figure 3a,b). Although the unequivocal identification and assignment of distinct peaks to specific chemical species of CN_*x*_ was nontrivial, three separated spectral regions could be identified. Namely, a first region from 3270 to 2700 cm^–1^ with a main peak at ∼3100 cm^–1^ (assigned to –NH and –NH_2_ stretching vibration), a second region from 2500 to 1770 cm^–1^, with a main bleach signal (defined here as a region with a lower extinction coefficient) at 2184 cm^–1^ (assigned to the characteristic C=N stretching of the cyanamide terminal group),^38^ and a third from 1700 to 880 cm^–1^, with the aromatic C–N stretching vibrations of the heptazine core.^49^

**Figure 3:**
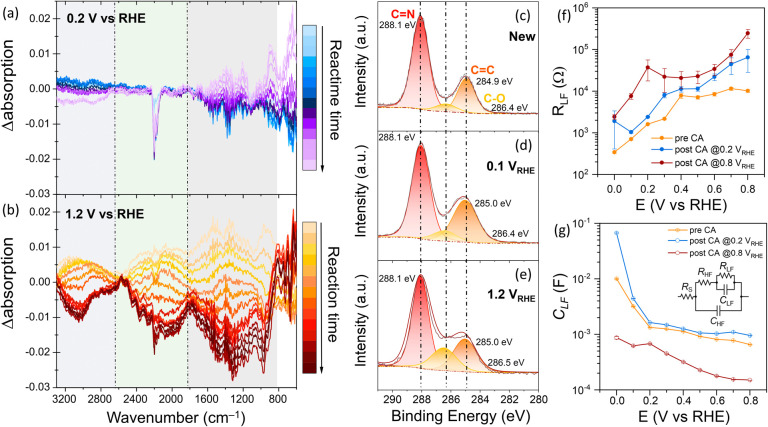
Spectroscopic analysis of the effect of light intensity and external applied potential on CN_*x*_/ITO photoanode stability. Difference (Δ) *in situ* synchrotron radiation-Fourier transform infrared (SR-FTIR) absorption spectra of the CN_X_/ITO photoanode under 1 sun illumination at (a) 0.2 V vs RHE or (b) 1.2 V vs RHE. SR-FTIR testing conditions: 3-electrode setup, Pt as counter electrode, Ag/AgCl (saturated KCl) as reference electrode, 50 mM glycerol in 0.1 M Na_2_SO_4_ (pH 7), customized 1-compartment SR-FTIR-PEC cell. The photoelectrode active area was 0.05 cm^–2^. Spectra were collected every 4 min over a total of 60 min. High-resolution XPS spectra of C 1s of (c) unreacted, (d) post 0.1 V, and (e) post 1.2 V vs RHE under 3 suns illumination (16 h) CN_*x*_/ITO photoanodes. Potential-dependent low-frequency (f) resistance (*R*_LF_) and (g) capacitance (*C*_LF_) values of CN_*x*_/ITO photoanodes pre-CAs (yellow trace), post 0.2 V vs RHE tests (20 h, blue trace), and post 0.8 V vs RHE tests (16 h, dark red trace) obtained from fitting the voltage-dependent Nyquist plot measured by PEIS before any CAs, and after holding the sample at 0.2 V vs RHE, and 0.8 V vs RHE, with a two-RC equivalent circuit (inset Figure 3g). PEIS conditions: 0.25 and 0.5 cm^–2^ electrode area, 1 compartment PEC cell (10 mL) in a 3-electrode configuration, Pt mesh as counter electrode, Ag/AgCl (saturated KCl) as reference electrode, 5 v/v % glycerol (0.68 M) in 0.1 M Na_2_SO_4_ (pH 7).

The difference absorption spectra at 0.2 V_RHE_ (Figure 3a) showed minimal changes in the absorption profile over time when compared to the one at 1.2 V_RHE_ (Figure 3b). In the former, the cyanamide functional group signal (2184 cm^–1^) displayed some bleaching within the first 8 min and then stabilized. In contrast, the absorption profile of the sample held at 1.2 V_RHE_ changed drastically over time. Even though the specific changes at a molecular level are challenging to assign, the general trend reflects a bleach in all three preidentified spectral regions characteristic of both the heptazine core (1700 to 880 cm^–1^) and surface functional groups of CN_*x*_. This trend indicates a gradual degradation of the chemical species associated with the CN_*x*_ material.

Control IR spectra collected in the absence of light, applied bias, or both (Figures S13 and 14 with extended discussion) showed minimal changes in absorbance over time, suggesting that the photoanode is stable in the electrolyte and that light alone does not strongly affect the sample morphology or surface chemical nature of the CN_*x*_, supporting PEC results.

Since self-oxidation of CN_*x*_ is considered one of the most probable degradation pathways,^32^ post-PEC X-ray photoelectron spectroscopy (XPS) analysis was conducted after 16 h CA at different light intensities and potentials, and compared to the fresh electrode (Figure 3c–e). The high-resolution C_1s_ of the untested sample was deconvoluted into three characteristic peaks assigned to C=N (288.1 eV), C=C (284.9 eV), and C–O (286.4 eV) bonds in the heptazine ring (Figure 3c), consistent with previous studies.^13^ While the photoanode subjected to 0.1 V_RHE_ showed only a minimal increase in the C–O peak (∼15%, Figure 3d), a marked 85% increase in the C–O component was observed after applying 1.2 V_RHE_ (Figure 3e). Under 1 sun, even greater C–O increases and shifts in C=N (288.4 eV) and C=C (285.2 eV) peaks were present (Figure S15). It is thus apparent that working under concentrated solar light and low applied potential minimizes severe surface oxidative degradation compared to more traditional operating conditions. However, oxidative degradation is predominantly potential-driven, rather than solely dependent on light intensity. The high-resolution O 1s spectra further confirm the presence of C–O on the surface of the photoanodes (Figure S16), while N 1s (Figure S16), In 3d, and Sn 3d spectra remained unchanged (Figure S17), indicating CN_*x*_ is the only component affected.

To further investigate the effect of the applied potential and light-dependent surface chemical changes to the CN_*x*_ electrochemical properties, potential-dependent PEC impedance spectroscopy (PEIS) analysis was conducted before and after performing CA under 3 suns at either 0.2 or 0.8 V_RHE_ for at least 15 h (5 v/v % glycerol 0.1 M Na_2_SO_4_, Figure 3f,g, Figure S18). The potentials were selected since the former lies within the previously identified stability window for the CN_*x*_ photoanode, while the latter exceeds the degradation threshold (∼0.25 V_RHE_), allowing direct comparison of electrochemical behavior under stable and unstable operations.

To thoroughly evaluate the electrochemical changes occurring to the CN_*x*_, the impedance response was fitted with a two-RC equivalent circuit (inset in Figure 3g), giving one high-frequency (*R*_HF_ and *C*_HF_), assigned to highly resistive electron transfer from the ITO particles to the FTO, and one low-frequency semicircle, assigned to electron extraction processes from CN_*x*_, as previously studied (Figure S18).^13^ The analysis was focused on the CN_*x*_-related low-frequency resistance (*R*_LF_) and bulk capacitance (*C*_LF_), which were found to be strongly influenced by the applied potential. Interestingly, *R*_LF_ always increased after CA tests, regardless of the applied potential (Figure 3f), as observed in other PHI-based electrodes.^28^ However, the applied potential greatly influenced the degree of rise in resistance. More specifically, the increase in *R*_LF_ was, on average, three times greater at 0.8 than at 0.2 V_RHE_. In contrast, the *C*_LF_ showed opposite trends. The sample held at 0.2 V_RHE_ showed a moderate ∼20% increase in capacitance, while a sharp ∼78% decrease occurred in the sample tested at 0.8 V_RHE_ (Figure 3g). Thus, the irreversible changes occuring at high applied potentials directly affect the CN_*x*_ electron transport properties and capability of holding charges. In contrast, under 1 sun, the applied potential had less influence on the capacitance loss, with over 70% decrease regardless of potential (Figure S19 with extended discussion). Thus, it is apparent that concentrated solar light is fundamental in ensuring long-term stability when subjecting the samples to low applied potentials, resulting in minor CN_*x*_ capacitance losses over time.

### Mechanistic Interpretation of Stability Enhancement

Our results demonstrate that the stability of cyanamide-functionalized CN_*x*_ photoanodes strongly depends on both applied potential and light intensity, pointing out that the CN_*x*_/ITO photoanode undergoes irreversible chemical and electrochemical surface changes above a threshold potential of ∼0.25 V_RHE_. Such degradation is likely initiated and confined at the electrode surface, as indicated by bulk characterization techniques (PXRD and SEM, Figures S20 and S21), which show no major postreaction structural changes. *In situ* PEC-IR spectroscopy provides direct evidence that high applied potentials induce progressive attenuation of vibrational features associated with both the cyanamide terminal groups (NCN) and the heptazine framework (Figure 3a,b). The early loss of the NCN signal suggests that terminal functionalities are particularly susceptible to oxidative degradation. This is further supported by complementary XPS analysis, revealing a pronounced increase in surface C–O species accompanied by a decrease in the relative C=N contribution (Figure 3c–e). These trends indicate that C–O bond formation likely originates from oxidative modification of C=N environments at the surface, in line with experimental and theoretical studies on polymeric carbon nitride.^32,50^

Such potential-driven surface changes are expected to diminish the oxidation capability and photoactivity of the material, accelerating the mechanical and electrical breakdown of the photoanode, consistent with the observed increase in resistivity and loss of capacitance at high applied potentials.

However, the stabilizing role of working under concentrated light appears less straightforward. Notably, as previously pointed out, the photocurrent does not scale linearly with light intensity, indicating that a significant fraction of additional photogenerated carriers is not effectively extracted. Since the glycerol oxidation photocurrent under 3 suns remains lower than that obtained with other selected substrates (e.g, 4-MBA) at 1 sun, the conductivity of the ITO binder is unlikely to be the limiting factor under these conditions.^13^

One possible explanation involves the saturation of intrabandgap states of ionic carbon nitride photoelectrodes under high light intensities,^33,34,51^ as previously reported.^52^ On the other hand, the near-quantitative FE suggests that the additional photogenerated holes are consumed by a non-Faradaic process. We therefore propose that these shallow trapped electrons can act as recombination centers for photogenerated holes, thereby reducing the steady-state hole density. Such a redistribution of charge carriers would suppress the accumulation of highly oxidizing species, likely responsible for CN_*x*_ self-oxidation, consistent with the mitigated capacitance loss observed under 3 suns at low applied potentials (Figure 3g).

Importantly, this stabilizing effect is lost once a sufficiently positive bias is applied (>0.25 V vs RHE), suggesting that external potential facilitates the extraction of these trapped electrons, thereby reducing the effect of working under concentrated light, leading to hole accumulation and promoting oxidative chemical degradation. The presence of a stable operating window below ∼0.25 V vs RHE may therefore not be governed by substrate oxidation kinetics, but by how applied bias interacts with light-induced charge trapping and recombination processes at the CN_*x*_ surface.

Although this fundamental study offers a first qualitative insight into the PEC CN_*x*_ degradation processes, further studies will be required to fully elucidate the interplay between the underlying charge carrier dynamics, surface photodegradation, and electrochemical stability under different light intensities and applied potentials.

Nevertheless, unlike most strategies so far focusing on improving the stability of CN_*x*_ photoelectrodes by incorporating cocatalysts,^17^ protective overlayers,^32^ or forming heterojunctions,^21,53^ this work adopts a fundamentally different approach. We have assessed the long-term performance of CN_*x*_-based photoelectrodes systematically by examining the influence of applied potential and light intensity, two critical yet overlooked parameters in CN_*x*_ photoelectrode development. The benchmark stability achieved in this work demonstrates that tuning operational conditions can be just as effective, if not more so, than structural modifications. This highlights the importance of re-evaluating standard PEC testing protocols and considering a broader range of experimental variables beyond pH and electrolyte composition when assessing the performance and durability of emerging photoelectrodes.

### TEG-PEC Device for Paired Photoelectrolysis

To take full advantage of working under concentrated solar light and low applied potential and to demonstrate the relevance of our findings for bias-free device operation, we assembled a two-compartment two-electrode TEG-PEC device for unassisted PEC glycerol oxidation and CO_2_-to-CO conversion (Figure 1). A heat-harvesting TEG unit was introduced between the heated photoanode and a room-temperature element (a chiller plate) to convert the local heat into additional internal voltage, allowing for efficient thermal and power management.^54,55^ A custom 3D-printed cell was designed to induce a temperature difference between the two sides of the TEG (details in Supporting Information, Figure S22). A bipolar membrane was employed to prevent substrate/product crossover (Figure S23).

To produce a uniform 1 cm^2^ CN_*x*_/ITO film for the photoanode, we replaced the optimized drop-casting method with spray coating. This technique can provide reproducible and scalable coatings, making it a superior method for depositing materials over larger areas compared to drop casting, which is more suited to small-scale or localized applications.^56^ Although CN_*x*_ is not soluble in most common solvents, ultrasonication in polar solvents (i.e., ethanol) is widely employed to yield a homogeneous quasi-solution of polymeric carbon nitride for photocatalytic applications.^57^ We employed a short 5 min ultrasonication to homogenize the heterogeneous CN_*x*_/ITO mixture and prevent sedimentation during spray coating, enabling the reproducible deposition of uniform films over a 1 cm^2^ area. By tailoring the composition, the spray-coated photoanodes matched the current density, thickness, and morphology of the drop-cast electrode (Figure S24). Top-view SEM and EDX mapping images show uniform film thickness of 3 ± 0.3 μm consisting of homogeneous blending of ITO nanoparticles within the CN_*x*_ matrix (Figures 1d and S25). As cathode material, a previously optimized Cu_97_In_3_ alloy was selected to reduce CO_2_ into CO with 70% FE at a low overpotential (onset potential approximately −0.2 V_RHE_).^58^ The Cu-based alloy was synthesized followinga template-assisted electrodeposition method (details in Supporting Information) and characterized by TEM and LSVs (Figures 1c and S26).

The assembled CN_*x*_/ITO|TEG||Cu_97_In_3_ device performance was first assessed by cyclic voltammetry under 3 suns (Figure 4a). As anticipated by the current crossover analysis of the LSV scans of the individual cathode and photoanode in a three-electrode configuration (Figure S27), the two-electrode PEC device could operate in bias-free mode even without the TEG wired, showing ∼80 μA cm^–2^ of photocurrent at 0 V bias voltage. Wiring the TEG in series introduced a 300 mV cathodic shift (onset: −0.6 V), consistent with the results obtained testing the cell operation with Pt as H_2_-evolving cathode (Figure S28 and extended discussion), reaching 190 μA cm^–2^ (forward scan) at 0 V bias voltage. This additional voltage is derived from the temperature difference between the TEG hot front and its cold backside in direct contact with the chilling unit at 20 °C, confirmed by infrared thermal images (Figure S29). We note that, under these modest temperature differences (∼20 °C), the thermal-to-electrical conversion efficiency of the commercial TEG unit is intrinsically low (<2%).^59^ However, beyond harvesting otherwise unused thermal energy, the TEG serves to generate enough bias to study the stability window identified in three-electrode measurements under device-relevant conditions.

**Figure 4:**
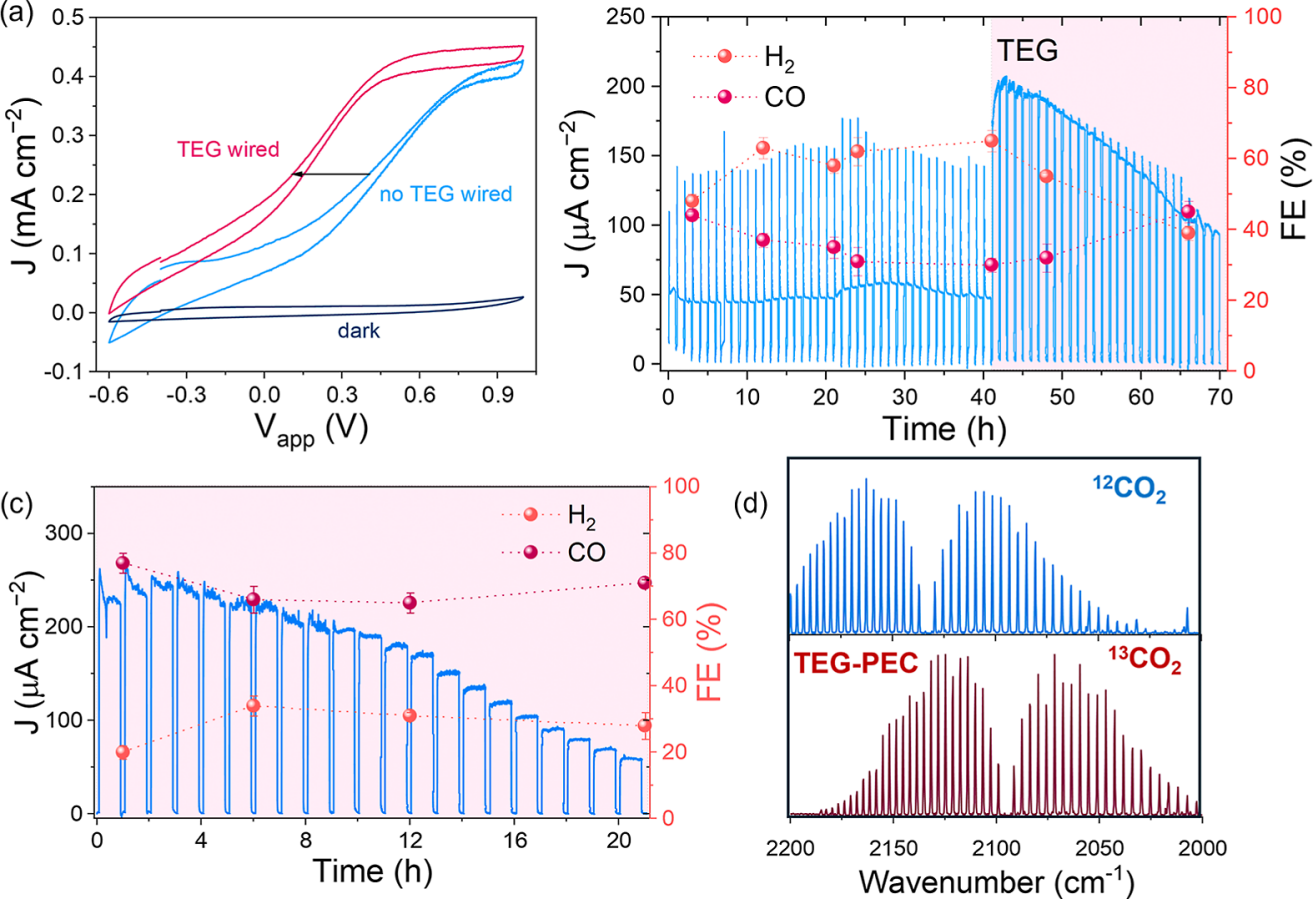
Analysis of PEC CN_*x*_/ITO|TEG||Cu_97_In_3_ device. (a) Cyclic voltammetry scans (10 mV s^–1^) of the CN_*x*_/ITO|TEG||Cu_97_In_3_ PEC setup in the dark (dark blue trace) and under 3 suns illumination without (blue trace) and with (dark pink trace) the wired TEG unit. (b) CA of the CN_*x*_/ITO|TEG||Cu_97_In_3_ device over 70 h chopped light without and with the TEG unit wired to the system, with H_2_ and CO FE measured over time. (c) 21 h CA of the CN_*x*_/ITO|TEG||Cu_97_In_3_ device with the TEG wired since the start of the measurements, with H_2_ and CO FE measured over time. The sample was stabilized for 30 min under constant illumination to let the electrolyte solution temperature increase and take advantage of the Seebeck effect. (d) Transmission IR spectra with ^13^CO_2_ isotopic labeling of the cathode headspace after CA tests (red spectrum), including ^12^CO (blue) as reference. Conditions: 3 sun-simulated solar light, two-electrode configuration, two-compartment 3D-printed PEC-TEG cell, 5 v/v % glycerol (0.68 M) 0.1 M Na_2_SO_4_ as anolyte (1.5 mL) purged with N_2_ for at least 20 min, 0.5 M NaHCO_3_ as catholyte (pH 7.2, 2.5 mL) purged with CO_2_ for at least 20 min, TEG connected in series, temperature as high as 40 °C under 3 suns.

The long-term PEC device stability was first studied with the TEG mounted but not wired (Figure 4b). The unbiased device could deliver an average photocurrent of 50 μA cm^–2^ for 41 h under chopped 3 suns illumination. The reduction products were analyzed by gas chromatography (GC), systematically sampling the cathode headspace. Starting from a 1:1 mixture of the two gases at early times (3 h), a 2:1 mixture of H_2_/CO was measured after 41 h of operation (FE of 68 ± 2% for H_2_ and 31 ± 3% for CO, corresponding to a total of 20.2 ± 0.9 μmol of H_2_ and 9.3 ± 0.6 μmol of CO).

Once the TEG was connected after 41 h, the current increased sharply, reaching a maximum of 200 μA cm^–2^. Then, the photocurrent starts decreasing slowly, maintaining 50% of the maximum activity (100 μA cm^–2^) after another 30 h of constant operation. This instability is only present when the TEG is wired to the device, and may be related to the additional voltage provided by the TEG (∼0.3 V) that is experienced by the CN_*x*_ photoanode. As discussed above, the photoelectrode becomes unstable above a specific threshold potential (∼0.25 V vs RHE) because of irreversible changes to its morphology and electronic properties. The response observed upon TEG integration in the PEC device therefore corroborates, under practical two-electrode conditions, the empirically identified stability window previously established through independent electrochemical, spectroscopic, and impedance analyses in a three-electrode configuration. In this context, the integration of the TEG unit into the final PEC device serves as a validation tool confirming that the stability parameters uncovered in the fundamental photoelectrochemical analysis could also be translated into an integrated, bias-free device architecture. By contrast, the Cu alloy was not affected by the experimental conditions, as confirmed by high-resolution TEM analysis (Figure S30).

TEG wiring also influenced product selectivity: FE shifted from ∼2:1 H_2_/CO to almost a 1:1, with 45% CO FE and 39% H_2_ FE (corresponding to a total of 34.6 ± 1.7 μmol of H_2_ and 40.0 ± 2.6 μmol of CO). The TEG is fundamental in providing enough voltage to allow the Cu_97_In_3_ to perform CO_2_-to-CO conversion over H_2_ evolution, as shown by experiments with the TEG connected from the beginning (Figure 4c). Namely, a constant 1:2 ratio between H_2_ and CO was detected, corresponding to 69 ± 5% FE for CO and 28 ± 6% FE for H_2_, a selectivity comparable to the literature.^58^ In contrast, the oxidation product selectivity remained constant throughout all measurements and is consistent with previous PEC tests (FE over 95% confirmed by HPLC analysis, Figures S31 and S32). The consistency under different illuminations, applied potentials, and reactor configurations suggests that glycerol oxidation on the CN_*x*_-based photoanode proceeds via an intrinsic surface-controlled mechanism, such that variations in operating conditions do not alter the dominant reaction pathway. The off/on TEG long CA measurements were performed in duplicates (Figure S33).

Isotopic labeling with ^13^CO_2_/NaH^13^CO_3_ confirmed no compartment crossover through the membrane. An analysis of the catholyte gas headspace by transmission IR spectroscopy showed the formation of only ^13^CO (Figure 4d). No reduction products were detected in exclusion-control experiments in the absence of light or CN_*x*_ (Table S1), confirming that the overall CN_*x*_/ITO|TEG||Cu_97_In_3_ device is driven by the CN_*x*_ photoabsorber. Control experiments were also performed at different light intensities. Under 1 sun, wiring the TEG had little effect due to minimal temperature difference between the TEG walls, and CA showed rapid current decay (>90% loss in 16 h, Figure S34). The same instability was observed under 4 and 5 suns, caused by a combination of thermal and applied potential-related degradation of the photoanode (Figures S35 and S36).

To our knowledge, the CN_*x*_/ITO|TEG||Cu_97_In_3_ system is not only the most stable PHI-based PEC device to date (Figure S37 and Table S2), performing continuously for 71 h, but it is the first reported PEC device where a CN_*x*_/ITO photoanode - metal alloy cathode assembly can drive simultaneous alcohol oxidation coupled to CO_2_-to-CO conversion without bias voltage. Moreover, this TEG-PEC device can be considered as a first step toward the scalable deployment of PEC technologies.^6^ It meets several fundamental criteria necessary for large-scale applications, including the use of earth-abundant materials for the photoelectrode, operation at neutral pH to enhance long-term stability and reduce costs, and fabrication via low-cost, scalable techniques.

Extending operation times and scaling up the device to larger areas are inherently linked challenges, both of which will require improved thermal and mass transport management. In particular, product accumulation and diffusion limitations under batch conditions are expected to become increasingly limiting at longer operation times and larger reactor dimensions.^6^ Transitioning to continuous-flow operation is therefore anticipated to be a critical next step, simultaneously enabling sustained long-term stability, improved reaction control, and scalable device architectures.

The integration of a TEG is fundamental to further optimizing thermal management, utilizing excess heat to provide additional voltage and improve overall efficiency.^36^ While the thermal-to-electricity conversion efficiency of current TEG modules remains modest, ongoing advances in thermoelectric materials, now approaching efficiencies of 15%, are expected to facilitate the maintenance of stable temperature gradients over larger areas and reduce the overall costs of the device.^60^ Nevertheless, PEC systems are inherently voltage-limited rather than power-intensive, such that even modest-efficiency TEGs can already provide functionally relevant bias, as this study shows.

Collectively, this work provides a basis for the future development of carbon nitride, and other semiconductor powders more broadly, in PEC devices for sustainable fuel and chemical production.

## Conclusions

Our work shines light on the critical impact of light intensity and applied potential on the long-term stability of CN_*x*_-based photoelectrodes. By moving beyond conventional PEC testing protocols, we demonstrate that operating under concentrated solar light and at low or no external bias significantly enhances photoanode stability. This approach enabled continuous, unbiased PEC operation for over 70 h (∼ 3 days) without cocatalysts or additional light absorbers, a benchmark for CN_*x*_ materials. Our findings show that the commonly used testing conditions at 1.2 V_RHE_, even in the presence of hole scavengers, accelerates degradation. *In situ* PEC-SR-FTIR revealed pronounced surface chemical changes at high potentials, absent when operating at <0.4 V_RHE_. These irreversible alterations, confirmed by XPS, likely increase CN_*x*_ susceptibility to self-oxidation. Additionally, PEIS analysis showed that CN_*x*_ photodegradation results in higher resistivity and reduced capacitance. By coupling the stabilized CN_*x*_/ITO photoanode with a Cu_97_In_3_ cathode in a two-compartment PEC device, we demonstrated a standalone system employing a CN_*x*_ photoelectrode to sustain alcohol oxidation coupled to CO_2_-to-CO conversion. The integration of a TEG enabled efficient solar thermal harvesting, supplying the extra voltage needed to favor CO_2_ reduction over proton reduction. The results reported herein showcase how CN_*x*_, an inexpensive semiconductor made from earth-abundant materials, can be engineerd to replace state-of-the-art metal oxides and other semiconductors in PEC devices in the future.

## Supplementary Material



## Data Availability

Data that support the findings of this study are available from the University of Cambridge data repository: 10.17863/CAM.127161.

## Notes

Materials and methods for photoanode synthesis, PEC testing, and TEG-PEC reactor assembly, PEC analysis,*in situ* FTIR, and EIS measurements as a function of light intensity and applied potential; UV–vis, XPS, EIS, SEM, and TEM characterization; infrared thermal imaging of the reactor; ICP-OES analysis; product quantification and characterization by NMR and HPLC (Figures S1–S37, Tables S1 and S2) (PDF)

## †

Samuel J. Cobb—Department of Chemistry, The University of Manchester, Manchester M13 9PL, U.K
