# Supplementary material for: Stabilizing Carbon Nitride Photoanodes for Unassisted Alcohol Reforming Coupled to CO2 Reduction under Concentrated Sunlight
Source: J Am Chem Soc. 2026 Mar 17;148(12):12839–48. doi: 10.1021/jacs.5c20624 (PMC13047678; doi:10.1021/jacs.5c20624)
Supplement: Supplementary file 1 [file ja5c20624_si_001.pdf]

Supporting information

Stabilizing carbon nitride photoanodes for unassisted alcohol reforming coupled to CO_2 reduction under concentrated sunlight

Carolina Pulignani,[1] Ariffin Bin Mohamad Annuar,[1] Samuel J. Cobb,[1] Motiar Rahaman,[1] Yongpeng Liu,[1] Chen Han,[1] Andrea Rogolino,[1] Subhajit Bhattacharjee,[1] Erwin Reisner[1,*]

[1]Yusuf Hamied Department of Chemistry, University of Cambridge, Cambridge CB2 1EW, UK

*E-mail: reisner@ch.cam.ac.uk

Experimental Section

Materials and chemicals. The chemicals and materials were purchased from commercial suppliers and used without further purification: N_2 gas bottle (2% CH_4 as internal standard, BOC), CO_2 gas bottle (2% CH_4 as internal standard, BOC), 4-methylbenzyl alcohol (4-MBA, Sigma-Aldrich, 98%), 4-methylbenzaldehyde (p-tolualdehyde, Thermo Scientific Chemicals, 99+%), melamine (Sigma-Aldrich, 99%), potassium thiocyanate (KSCN, Sigma-Aldrich, ≥99.0%), sodium sulphate (Na_2SO_4, Sigma-Aldrich, ≥99.0%), glycerol (Sigma-Aldrich, ≥99.0%), glyceraldehyde (Sigma-Aldrich, ≥90%), dihydroxyacetone (Sigma-Aldrich, ≥98%), ethanol (Sigma-Aldrich, 96%), fluorine-doped tin oxide (FTO, ~7Ω sq^{-1}) coated glass, indium tin oxide (ITO) nanoparticles (<50 nm diameter, surface area 47 m^2 g^{-1}, Sigma Aldrich), ^{13}C-$NaHCO_3$ (Sigma Aldrich), $^{13}CO_2$ (Sigma Aldrich), Na_2CO_3 (Sigma Aldrich), KCl (Sigma Aldrich), and $NaHCO_3$ (Sigma Aldrich). Copper(II) sulfate pentahydrate ($CuSO_4 \cdot 5H_2O$, 99.995%), indium(III) sulfate hydrate ($In_2(SO_4)_3 \cdot xH_2O$, 99.99%), and Suprapur sulfuric acid (H_2SO_4, 96%) were purchased from Sigma Aldrich, Cu foil (99.9%) from Alfa Aesar, orthophosphoric acid (H_3PO_4, 68%) was purchased from Fluka. ACS reagent grade $NaHCO_3$ (≥99.7%) was purchased from Sigma Aldrich. Matte black spray paint (Simply Sprays), thermal grease (CTCM78-1, 7.8 W mK^{-1}, Conrad), Cu foil (1.0 mm, 99.99% metal basis, Fisher). MilliQ H_2O (18.2 MΩ cm) was used for all the experiments.

Carbon nitride powder synthesis. Melamine-derived CN_x was synthesized following a previously reported procedure.[1] 5 g of melamine was heated up at 550°C in a crucible with a lid for 3 hours at a ramp rate of 1°C min^{-1}. The recovered pale-yellow powder (2.5 g) was ground finely with a mortar and pestle before post-functionalization. The cyanamide-functional group was introduced by grinding together 1.5 g of the freshly synthesized CN_x with 3 g of potassium thiocyanate.[2] The powder mixture was placed into a ceramic boat and heated up at 400 °C for an hour and then at 500°C for 30 min at a ramp rate of 30 °C min^{-1} under inert atmosphere (argon). The bright yellow powder was allowed to cool down to room temperature inside the oven, and then washed twice with D.I. water and once with a mixture of water and ethanol. The clean photocatalyst powder was dried overnight at 50 °C before being finely ground with a mortar and pestle.

CN_x/ITO Photoanode synthesis: drop-casting. Pre-cut FTO-coated glass slides of 1 x 3 cm were cleaned by sonication in isopropyl alcohol, followed by ethanol (25 min each), dried under nitrogen, and cleaned in a UV ozone generator for 15 min. CN_x-based photoanodes were synthesized following a previously optimized procedure.[3] The carbon nitride powder was mixed with an equal amount of commercial ITO nanoparticles (diameter < 50 nm, surface area

47 $m^2 g^{-1}$) and sonicated in ethanol for 30 min (25 mg in total in 550 µL). The deposition area of the electrode was delimited with a mask prepared either by cutting Parafilm with a drilling bill (0.25 or 0.5 cm^{-2} circle). The Parafilm template was pressed onto the FTO-coated side of the clean glass slides with the help of plastic tweezers and slightly heated (30 s in a 120°C drying oven) to ensure a uniform adhesion of the Parafilm mask onto the conductive substrate. Two layers of 5 µL were drop-cast inside the template. The mask was carefully removed before annealing the photoelectrodes at 250°C for 2 h under Ar (ramp rate 10°C min^{-1}). Please refer to reference *3* for detailed statistical analysis and structural metrics, parameters such as film thickness, morphology, crystallinity, compositional uniformity, and detailed PEC analysis.

CN_x/ITO Photoanode synthesis: spray-coating. For the synthesis of the 1 cm^{-2} photoanode employed in the TEG-PEC device, a solution of the previously synthesized cyanamide-functionalized carbon nitride and commercial ITO nanoparticles were combined in a 1:1 weight ratio (8, 16, 20, or 24 mg in total), dispersed in ethanol (1 mL) ultrasonicated for 5 min (30 sec on/10 sec off, 50% power) and then sonicated for 30 min. 200 µL of the mixture was spray-coated onto a 1 cm^2 templated (Kapton tape masked) FTO glass at 40ºC with a Paasche HS-202S Single Action Siphon Feed Airbrush (20 psi, 3 sec on/ 3 off, 8 cm distance) for 3 sec, let dry for another 3 sec to 5 sec, and sprayed again until the area was homogenously covered. Once dried, the Kapton tape mask was removed, and the electrodes were annealed at 250°C for 2 h under Ar (ramp rate 10°C min^{-1}).

Preparation of $Cu_{97}In_3$ catalyst. The CuIn alloy was synthesized via template-assisted electrodeposition, following a previously reported method.[4] Cu foil, electropolished in 50% ortho-phosphoric acid (+2.0 V for 90 s), was used as substrate for electrodeposition. The electrodeposition was conducted in a clean glass beaker containing copper sulfate and indium sulfate precursor salts (0.025 M) in a 1.5 M sulfuric acid solution. The deposition was performed in a three-electrode set-up where Cu foil was used as the working electrode, a Cu foil (4 x 4 cm) as the counter electrode, and a leak-free Ag/AgCl (saturated NaCl, BASI) electrode as the reference electrode. A current density of $J = -3.0$ A cm^{-2} was applied for 60 s. The porous architecture was achieved thanks to the co-produced H_2 bubble, which acts as a template for the alloy during the electrodeposition. After the galvanostatic deposition, the catalyst was cleaned by dipping it into Milli-Q water for 120 s and dried under N_2 stream at room temperature.

CN_x/ITO|$Cu_{97}In_3$ TEG-PEC device assembly. A thermoelectric cooler module (Adaptive Peltier Module, 21.2W, 3.9A, 8.8V, 20 x 20mm) was first attached with grey thermal paste to

the aluminum wall of a cooling block (2.5 x 2.5 cm) connected to a chiller to maintain a temperature of 20˚C on the cold side of the TE module. The back side of the CN_x/ITO photoanode (glass side of the FTO-coated glass) was then attached to the thermoelectric using the same grey thermal paste. The TEG-PEC reactor was designed and 3D-printed with a FormLabs 3B printer (SLA technology). All parts were made of a Rigid 10K Resin (88.0 MPa ultimate tensile strength, 11.0 GPa tensile modulus, and 158.0 MPa Flexural Strength), apart from the back closing block of the anode side that was made of Grey Pro Resin (61 MPa ultimate tensile strength, 2.6 GPa tensile modulus, and 86.0 MPa Flexural Strength). The device was assembled starting from the anode side, placing the commercial bipolar membrane between the tightened anode and the cathode compartments. The anode side was filled with 1.5 mL of 5 v/v % glycerol 0.1 M Na_2SO_4 electrolyte solution (pH 7), whilst the cathode compartment was filled with 0.5 M $NaHCO_3$ electrolyte solution. The compartments were sealed with septa and purged with N_2 and CO_2 gas, respectively, for at least 30 minutes.

3-electrode PEC oxidation tests. A single-compartment cell in a three-electrode configuration was filled with a 5 v/v % glycerol 0.1 M Na_2SO_4 electrolyte solution (pH 7). Ag/AgCl in saturated KCl was used as the reference electrode, and Pt (Pt mesh supported on a Pt wire) as the counter electrode. After purging the cell with N_2 (~15 min), linear sweep voltammograms (LSVs) and cyclic voltammograms (CVs) were performed by applying a potential from -0.2 to +1.6 V vs. RHE at a rate of 10 mV s^{-1} under chopped simulated solar light (AM 1.5G, 100 mW cm^{-2}, 10 s on/off intervals). Chronoamperometries (CAs) were performed at a constant applied potential vs RHE for more than 6 hours under chopped simulated solar light (AM 1.5G, 100 mW cm^{-2}, 50 min on/10 min off intervals). Unless otherwise mentioned, all the potentials have been converted to the RHE scale from the Ag/AgCl scale according to Eq 1.

$$E_{RHE} = E_{Ag/AgCl} + E^0_{Ag/AgCl} + 0.059 \times pH \qquad \text{Equation 1}$$

Where E_{RHE} is the potential versus RHE, $E_{Ag/AgCl}$ is the measured or applied potential versus Ag/AgCl, and $E^0_{Ag/AgCl}$ is the standard potential of the Ag/AgCl reference electrode. It is important to note that the value of $E^0_{Ag/AgCl}$ changes with temperature.[5] At 25ºC, the standard potential for the Ag/AgCl (saturated KCl) reference electrode is 0.197 V. The exact dependence of the standard potential on temperature can be expressed using Eq. 2:[6]

$$E^0_{\frac{Ag}{AgCl}}(T) = E^0_{\frac{Ag}{AgCl}}(25^{\circ}C) + (T - 25^{\circ}C) \times \frac{dE^0_{Ag/AgCl}}{dT} \qquad \text{Equation 2}$$

For the Ag/AgCl reference electrode, the temperature coefficient $\frac{dE^0_{Ag/AgCl}}{dT}$ is approximately –0.7 mV/°C for each degree Celsius change from 25°C.

So, at a temperature *T* different from 25°C, the potential of the Ag/AgCl reference electrode changes, and the adjusted Eq. 3 for $E^0_{\frac{Ag}{AgCl}}(T)$ was employed:

$$E^0_{\frac{Ag}{AgCl}}(T) = 0.197\,V - 0.0007 \times (T - 25) \quad \text{Equation 3}$$

Incorporating Eq. 3 in Eq. 1, the temperature-adjusted formula (Eq. 4) to convert the potential to the RHE scale from the Ag/AgCl scale is

$$E_{RHE} = E_{Ag/AgCl} + [0.197\,V - 0.0007 \times (T - 25)] + 0.059 \times pH \quad \text{Equation 4}$$

Eq. 4 was employed to accurately account for temperature variations when converting potentials from Ag/AgCl to RHE in PEC experiments under concentrated solar light and temperature-dependent controlled tests.

To focus on intrinsic stability trends, all PEC experiments were carried out under strictly O_2-free conditions, with electrolytes purged with N_2 for at least 20 min before measurement and operated in a gas-tight cell

All PEC measurements reported in the present manuscript were performed using at least three independently prepared electrodes, and the reported trends (photocurrent densities, stability behavior, and impedance responses) were consistently observed across all replicates.

Temperature-dependent PEC measurements of CN_x-based photoanodes. A single-compartment jacketed cell (18 mL total volume) in a three-electrode configuration (Ag/AgCl in saturated KCl was used as reference electrode and Pt (Pt mesh supported on a Pt wire) as the counter electrode) was filled with 10 mL 0.1 M Na_2SO_4 and the substrate of interest (50 mM 4-MBA or 5 v/v% glycerol), purged with N_2 for 30 min, and connected to a chiller to control the temperature. The cell was then heated to the experimental temperature (25 – 70°C) and allowed to equilibrate before a CV (from -0.2 to +1.6 V vs RHE), and 6 h chronoamperometry experiments were performed. The potential applied was corrected by the change in pH and the standard potential of the reference electrode with temperature.

CN_x/ITO|$Cu_{97}In_3$ TEG-PEC device testing. The PEC measurements were performed either with a LOT-QD LS0816-H large area solar simulator equipped with an Air Mass 1.5 Global (AM 1.5G) solar filter, or 150 W xenon arc lamp (LOT-Quantum Design GmbH, LSE140/160.25C), calibrated to AM 1.5G. The solar concentration was achieved with a

commercial Fresnel lens (XL Full Page, 3× magnification, and Ø2" Fresnel Lens, f = 51 mm). The light intensity was varied between 1 and 5 suns (i.e., 100-500 mW cm^{-2}) by adjusting the distance between the lens and PEC reactor, and measured with a Newport 843-R optical power meter. All measurements were recorded with an InviumStat potentiostat. The photoanode was singularly tested in a 3-electrode setup, with Pt mesh as counter electrode and Ag/AgCl as reference electrode. The anode side was filled with 1.5 mL of 5 v/v % glycerol 0.1 M Na_2SO_4 electrolyte solution (pH 7), whilst the cathode compartment was filled with 0.5 M $NaHCO_3$ electrolyte solution. The compartments were sealed with septa and purged with N_2 and CO_2 gas, respectively, for at least 30 minutes. The photoanode was then connected in series to the TE module and dark cathode. CV scans were recorded at 10 mV s^{-1} scan rate. Long-term two-electrode PEC experiments were conducted under chopped light (50 min on, 10 min off) with no external applied potential in the 2-electrode configuration.

Electron microscopy. Scanning electron microscopy (SEM) was conducted on a TESCAN MIRA3 FEG-SEM. Samples were sputter-coated with a 10 nm layer of Cr (for SEM coupled with energy-dispersive X-ray spectroscopy, or EDX) before measuring the samples. Catalyst powder was placed on conductive carbon tape before sputtering and measurement. Transmission electron microscopy (TEM) was conducted on a Thermo Scientific (FEI) Talos F200X G2 TEM. All samples were prepared by scratching the deposited catalyst mixture from the FTO glass post-annealing. The mixture was then dispersed in ethanol (low concentration), drop-cast onto carbon-coated Cu TEM grids, and allowed to dry before use.

Attenuated total reflectance Fourier transform infrared (ATR-FT-IR) spectroscopy. FT-IR spectra were recorded on a Thermo Scientific Nicolet iS50 spectrometer. The Omnic software was used for analysis.

In-situ synchrotron radiation (SR)-FT-IR spectroscopy. The *in-situ* synchrotron radiation-Fourier Transform Infrared spectrum (SR-FTIR) measurements were carried out in an Infrared Micro-spectroscopy beamline at the Australian Nuclear Science and Technology Organisation (Clayton, Melbourne). The PEC cell and setup were previously optimized and employed to study solar-driven ammonia production.[7] A homemade 3-electrode photoelectrochemical cell was employed, with a ZnSe window. The data were collected on an FTIR spectrometer (Bruker Vertex) with a liquid nitrogen-cooled narrow-band mercury cadmium telluride (MCT) detector, coupled with an IR microscope (Bruker Hyperion 3000) with a 20x objective. Each IR adsorption spectrum was acquired in a reflection mode by 512 scans at a resolution of 4 cm^{-1}. The background spectrum was collected before the PEC test. An optical fiber is used as the light source on the working electrode. A different ITO:CN_x electrode (0.05 cm^2 area) was used for each set of measurements at the two different applied potentials, in a three-electrode setup,

with Pt as counter electrode and Ag/AgCl as reference electrode in 50 mM glycerol in 0.1 M Na_2SO_4 aqueous solution (pH 7).

Ultraviolet-visible (UV-vis) spectroscopy. UV-vis spectra were recorded on a Varian Cary 60 UV-Vis spectrophotometer using a diffuse reflectance accessory (for powder and electrodes). The measured diffuse reflectance of the mixture is then inverted directly by the program using the Kubelka–Munk theory. The CN_x absorption (λ < 450 nm) is increased upon the introduction of the cyanamide functional group (λ < 460 nm), turning the powder semiconductor from pale yellow to orange.

X-ray photoelectron spectroscopy (XPS). XPS analysis was carried out using an Escalab 250XI spectrometer from Thermo Fisher Scientific (West Sussex, UK). The instrument was operating in constant analyzer energy mode. A monochromatic Al-Kα source (1486.74 eV), a flood gun for charge neutralization, and 300 μm spot size were used. Survey scans were acquired using a pass energy of 100 eV, and 3 scans were recorded using 0.5 eV step size and a dwell of 50 ms.

Powder X-ray diffraction (XRD). XRD was conducted on a Panalytical Empyrean Series 2 instrument using Cu K_α irradiation, and the peaks were identified by comparison to literature XRD patterns.

Photoelectrochemical impedance spectroscopy (PEIS). Voltage-dependent PEIS measurements were carried out in an electrochemical cell with a 3-electrode configuration: a CN_x-based working electrode, a Pt mesh counter electrode, and an Ag/AgCl reference electrode (3 M NaCl gel, 0.55 mm diameter ceramic frit, MW-2030, BASi). The measurements were recorded with a potentiostat (IviumStat) with frequency ranges from 1 MHz to 50 mHz and a 25 mV sinusoidal amplitude. Impedance data were fitted with equivalent circuits using modelling software ZView2 (Scribner Associates). The electrolyte (10 mL) contained 5 v/v % glycerol in 0.1 M Na_2SO_4 (pH 7). A 150 W xenon arc lamp (LOT-Quantum Design GmbH, LSE140/160.25C), calibrated to AM 1.5G (100 mW cm^{-2}) with a Newport 843-R optical power meter, was used as a light source, equipped with a Fresnel lens (RS components) to concentrate light up to 3 suns (i.e., 200-300 mW cm^{-2}). The first measurement was done in the dark, with the sample never being exposed to simulated solar light. The pre-CA impedance was then recorded, followed by CV scans and CA under chopped simulated solar light. Finally, the post-CA impedance response was recorded under dark and light conditions before measuring the CV scans.

Gas Chromatography. The evolution of H_2 and CO was analyzed by headspace gas analysis using a Shimadzu Tracera gas chromatograph (GC) fitted with a barrier ionization discharge

detector and Molsieve column with He as the carrier gas. Methane (2% in N_2) was used as an internal standard. Aliquots of 50 μL of the headspace gas were removed from the sealed photocatalytic vials or photoelectrode cell using a gastight syringe (Hamilton) for GC analysis.

High-performance liquid chromatography (HPLC). All analyses were conducted on a Waters Breeze system equipped with refractive index (RID-2414) and diode array UV–vis (λ = 210 and 254 nm) detectors. Glycerol oxidation products were analyzed with an Ion-Exclusion ROA-Organic Acid H+ (8%) column at 70°C. Samples were analyzed in the isocratic flow mode (flow rate 0.5 mL min^{-1}, 0.005 M H_2SO_4 HPLC water). Calibrations were conducted with external standards for all substrates. On the other hand, 4-MBA and the relative oxidation product (4-methylbenzaldehyde) were identified and quantified with a C18 column at 40 °C column temperature. Samples were analyzed in the isocratic flow mode (flow rate 0.5 mL min^{-1}, H_2O:MeCN 1:1).

Nuclear magnetic resonance (NMR). ^{1}H NMR spectroscopy was used to confirm the glycerol oxidation products and check the possible products and substrate crossover between anode and cathode in the TEG-PEC 3D-printed cell. NMR spectra were collected with a Bruker 400 MHz Neo Prodigy spectrometer at 25ºC, with deuterated water as solvent at room temperature. Chemical shifts for ^{1}H NMR spectra are referenced relative to residual protons in the deuterated solvent (Eurisotop), and 3-(trimethylsilyl)propionic-2,2,3,3-d_4 acid, sodium salt in D_2O was used as the internal standard (TSP).

Faradaic efficiency calculation. The Faradaic efficiency (FE) of the products formed was calculated using Eq. 5, where *Z* is the number of electrons transferred, *n* is the number of moles of product formed, *F* is the Faraday constant (96485 C mol^{-1}), and Q_{passed} is the total amount of charge passed during the same time interval.

$$FY[product](\%) = \frac{Z \times n \times F}{Q_{passed}} \times 100 \qquad \text{Equation 5}$$

Supporting Figures

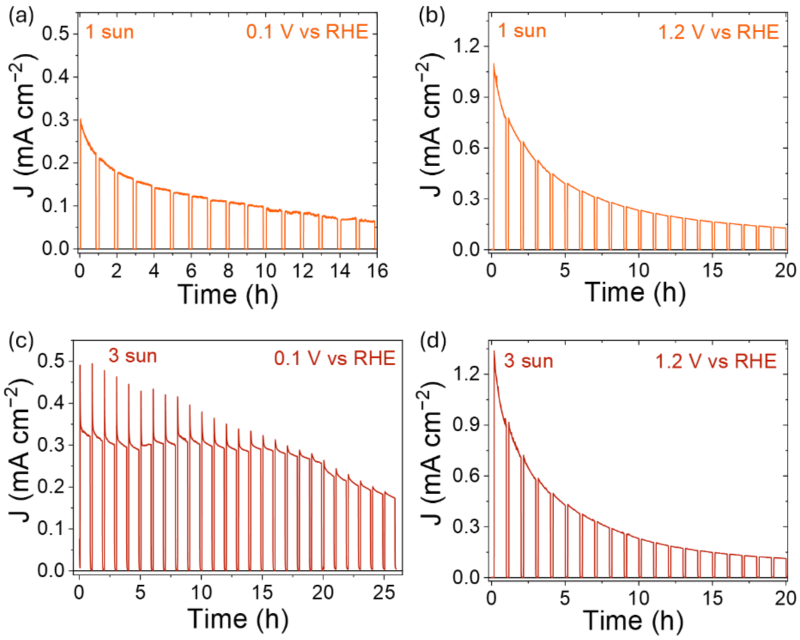

Figure S1. Chronoamperometry tests of CN$_x$/ITO photoanodes in a 3-electrode setup under 1 sun (a) 0.1 V vs RHE and (b) 1.2 V vs RHE. Chronoamperometry tests of CN$_x$/ITO photoanodes under 3 suns (c) 0.1 V vs RHE and (d) 1.2 V vs RHE. Conditions: 0.25 and 0.5 cm–2 electrode area, Pt mesh as counter electrode, Ag/AgCl as reference electrode, 5 v/v% Glycerol in 0.1 M Na2SO4 aqueous solution, chopped simulated solar light (air mass 1.5 G, 100 mW cm–2), 50 min on/10 min off for the CA measurements. The temperature was not controlled during the measurements, but it was measured before and at the end of the CA test. The temperature of the electrolyte solution reached 40 °C after 16 h under concentrated solar light (3 suns) and 28 °C under 1 sun. The pH did not change after any CA tests. Measurements were performed in triplicate.

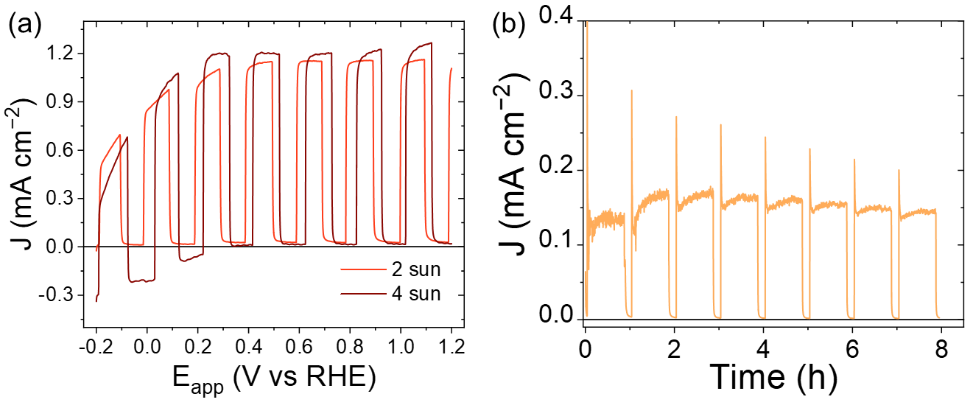

Figure S2. (a) Linear sweep voltammetry scans of CN$_x$/ITO photoanodes under 2 and 4 suns illumination in a 3-electrode setup. (b) chronoamperometry of the photoanode under 2 suns and 0.2 V vs RHE. Conditions: 0.25 and 0.5 cm^{-2} electrode area, Pt mesh as counter electrode, 5 v/v% glycerol in 0.1 M Na_2SO_4 aqueous solution, chopped simulated solar light (air mass 1.5 G, 100 mW cm^{-2}) 10 sec on/ 10 sec off for the CV scans (10 mV/s) and 50 min on/10 min off for the CA measurements. All measurements were performed in triplicate.

Extended Discussion Figure S2 on the effect of light concentration on the photocurrent response. One of the highest stabilities ever reported for PHI-based photoanodes was achieved under 2 sun illumination, in a two-electrode configuration at 0.5 V vs Pt, using methanol as an electron donor.[8] Although the application of concentrated light can accelerate photodegradation due to enhanced electron accumulation and radical formation—as noted by the authors—higher light intensity can also be beneficial by increasing substrate oxidation kinetics and boosting the overall photocurrent. In our case, however, the photocurrent response did not show a significant increase when moving from one sun to three suns; it remained around 0.9 mA cm^{-2} at 1.2 V vs RHE. This suggests that the reaction kinetics are not strongly dependent on light intensity, contrary to observations made for PHI photoanodes used for methanol oxidation.[8] Instead, these results are in line with previous spectroscopic studies on the CN_x/ITO photoanode, which indicated that performance is limited by the conductivity of the ITO nanoparticles.[3] As such, increasing the light intensity does not further improve the photocurrent, as it is constrained by the photoanode design itself.

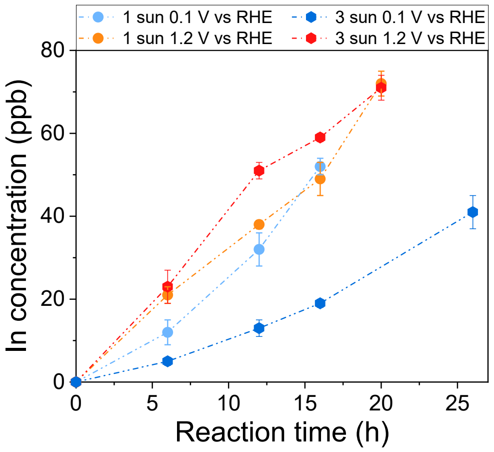

Figure S3. Inductively Coupled Plasma Optical Emission Spectroscopy (ICP-OES) of indium (from ITO nanoparticles of the CN_x/ITO photoanode) in the anolyte solution after CA tests at 0.1 V and 1.2 V vs RHE under either 1 or 3 suns-simulated solar light illumination. All measurements were performed in triplicate.

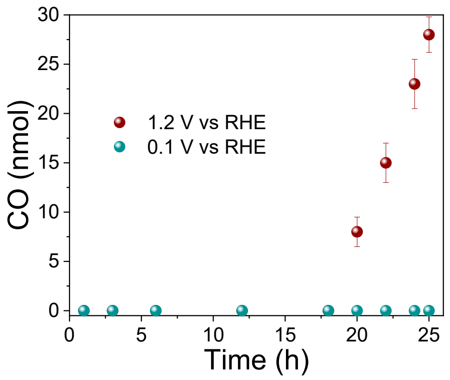

Figure S4*.* **CO evolved over 25 h CA.** Amount of CO evolved over time during CA measurements of CN_x/ITO photoelectrodes under 0.1 V and 1.2 V vs RHE in a 2-compartment PEC cell. Conditions: 1 and 3 suns illumination respectively, 5 v/v% glycerol in 0.1 M Na_2SO_4, 2-compartment PEC cell in 2-electrode configuration, Pt as counter electrode. CO measures through regular GC injections of the anode headspace. All measurements were performed in triplicate. It is important to note that CN_x have been shown to evolve CO when undergoing photodegradation, especially in a CO_2-rich atmosphere.[9]

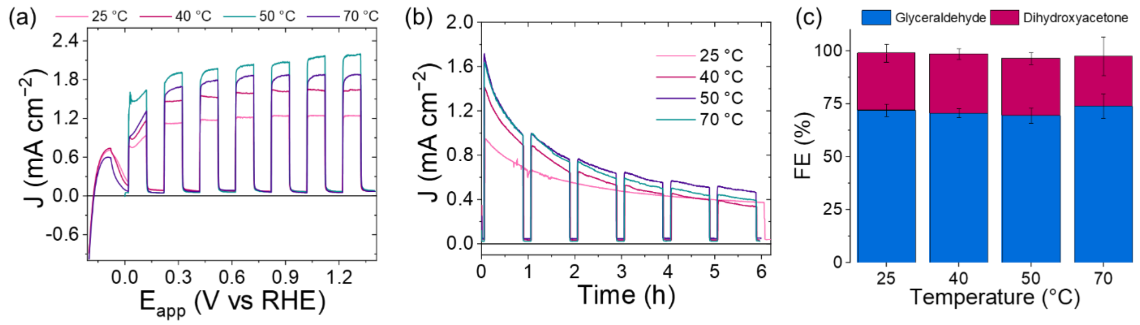

Figure S5. Temperature-controlled PEC studies. (a) Linear sweep voltammetry scans and (b) chronoamperometries of CN_x/ITO photoelectrodes at different temperatures: 25ºC (red trace), 40ºC (light blue trace), 50ºC (dark blue trace). (c) FE and product distribution of glycerol PEC oxidation after 6h CA at different controlled temperatures. Conditions: 1 compartment jacked PEC cell (10 mL) in a 3-electrode configuration, 5 v/v % glycerol in 0.1 M Na_2SO_4 (pH 7), 1 sun. CA performed at 0.1 V vs RHE. All measurements were performed in triplicate.

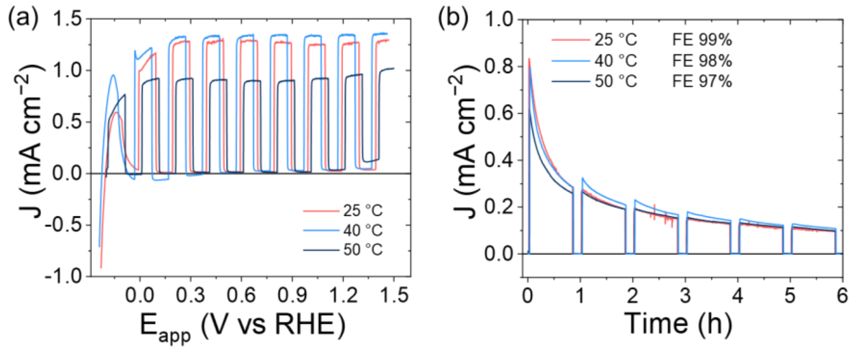

Figure S6. Temperature-controlled PEC analysis with 4-MBA. (a) Linear sweep voltammetry scans and (b) chronoamperometries of CN_x/ITO photoelectrodes at different temperatures: 25ºC (red trace), 40ºC (light blue trace), 50ºC (dark blue trace). Conditions: 1-compartment jacked PEC cell (10 mL) in a 3-electrode configuration, 50 mM 4-MBA in 0.1 M Na_2SO_4 (pH 7), 1 sun, CA under an applied bias of 0.1 V vs RHE. All measurements were performed in triplicate.

Extended Discussion Figure S6. The temperature-dependent PEC activity was recorded in a 1-compartment water-jacket cell in a 3-electrode configuration to decouple the effect of increasing light intensity from the concomitant local temperature increase on the photoelectrochemical oxidation kinetics. The temperature was varied from room temperature (25 ºC) up to 70 ºC, reachable under 10 suns operating conditions. These control experiments demonstrated that the local temperature increase from concentrated solar light is not the primary cause of improved long-term stability under low applied potentials. Long-term stability tests under chopped light and 0.1 V vs RHE showed no significant influence of temperature on the electrode performances. Notably, product selectivity remained unchanged across temperatures, maintaining a 2:1 ratio of dihydroxyacetone to glyceraldehyde.

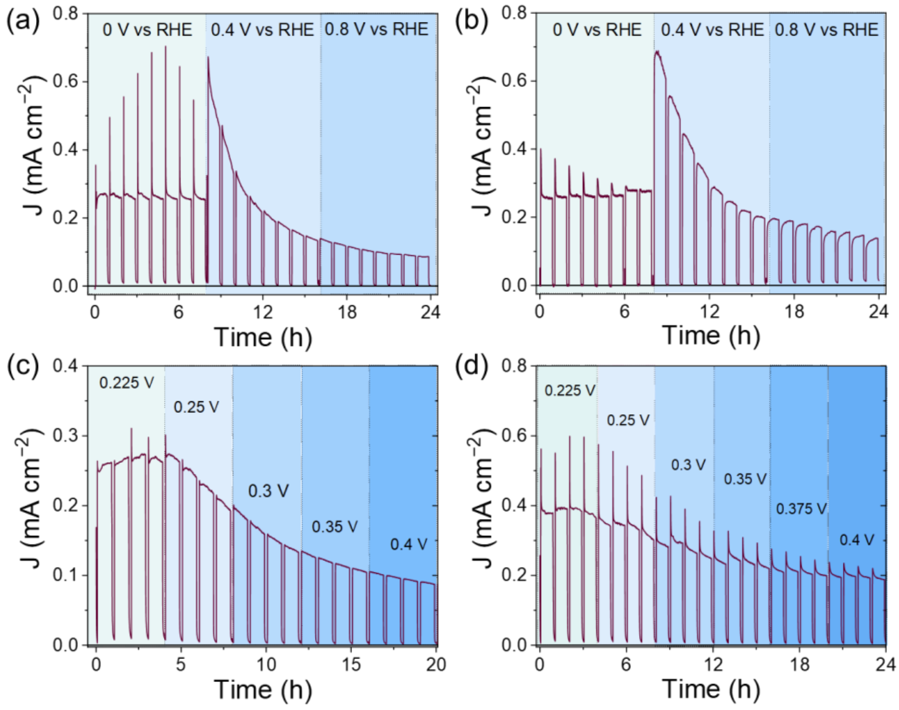

Figure S7. Effect of applied potential on photoanode stability. Chronoamperometries of CN_x/ITO photoanode under increasing external applied potential under (a,c) 2 and (b,d) 3 suns in a 3-electrode configuration. Conditions: 1 compartment PEC cell (10 mL) in a 3-electrode configuration, Pt mesh as counter electrode, Ag/AgCl as reference electrode, 5 v/v % glycerol in 0.1 M Na_2SO_4 (pH 7), 50 min light on/10 min light off. All measurements were performed in triplicate.

Extended Discussion Figure S7. To better understand the specific potential at which the photoanode becomes unstable, the samples were tested under 2 and 3 suns, increasing the applied voltage stepwise every 8 h. Figure S-7a and b show how by passing from 0 to 0.4 V vs RHE, the photocurrent previously stable for 8 h starts exponentially decaying. Even though the initial current value increases from 250 ± 15 μA cm^{-2} to 650 ± 10 μA cm^{-2} under 2 sun and from 275 ± 10 μA cm^{-2} to 680 ± 12 μA cm^{-2} under 3 sun after applying 400 mV extra, following the JVs scans, it reaches a lower value (135 ± 10 μA cm^{-2}) than the starting one at 0 V vs RHE (250 ± 15 μA cm^{-2}) after 8 h at 0.4 V vs RHE. Moreover, by applying an extra 400 mV, reaching 0.8 V vs RHE, the photocurrent does not increase but, on the contrary, it continues decreasing, reaching 86 ± 4 μA cm^{-2} under 2 sun and 130 ± 8 μA cm^{-2} under 3 sun, and it is not recoverable even by applying the first low voltage. From these results, the threshold potential at which the CN_x/ITO photoanode becomes unstable under concentrated solar light appears to be between 0 and 0.4 V vs RHE. Since previous results showed long-term stability at 0.2 V vs RHE, the electrodes were tested under 2 and 3 sun illumination, and the external applied bias was scanned from 0.225 V to 0.4 V with steps of 25 or 50 mV, to identify the instability threshold potential. As visible from Figure S-7c and d, the CN_x/ITO photocurrent starts degrading already at 0.25 V vs RHE, and increasing the applied potential does not correspond to an increase in current but rather to a constant decrease. As before, the initial photocurrent could not be recovered. All tests were performed for no longer than 24 h since previous results showed that the electrode can withstand 20 h of operation before degrading.

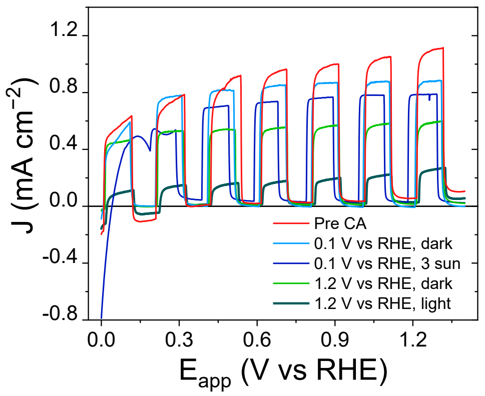

Figure S8. Linear sweep voltammetry scans of CN_x/ITO photoanodes (red) before 24h CA, after 24 h CA at 0.1 V vs RHE in (light blue) the dark and (dark blue) under 3 suns illumination, and after 24 h CA at 1.2 V vs RHE (light green) in the dark and (dark green) under 3 suns illumination. Conditions: 1 compartment PEC cell (10 mL) in a 3-electrode configuration, Pt mesh as counter electrode, Ag/AgCl as reference electrode, 5 v/v % glycerol in 0.1 M Na_2SO_4 (pH 7), chopped 3 suns simulated solar light 10 sec on/10 sec off. All measurements were performed in triplicate.

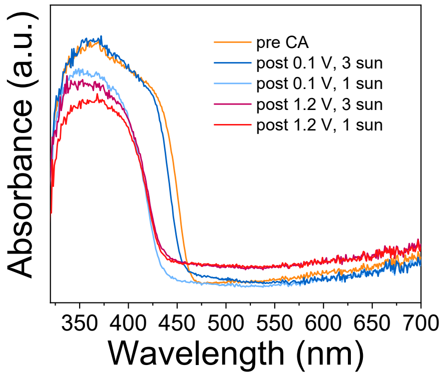

Figure S9*.* UV-Vis spectra of CN_x/ITO photoanodes (orange) before any CA tests, and after CA tests at 0.1 V vs RHE under 1 and 3 suns illumination, and 1.2 V vs RHE under 1 and 3 suns illumination.

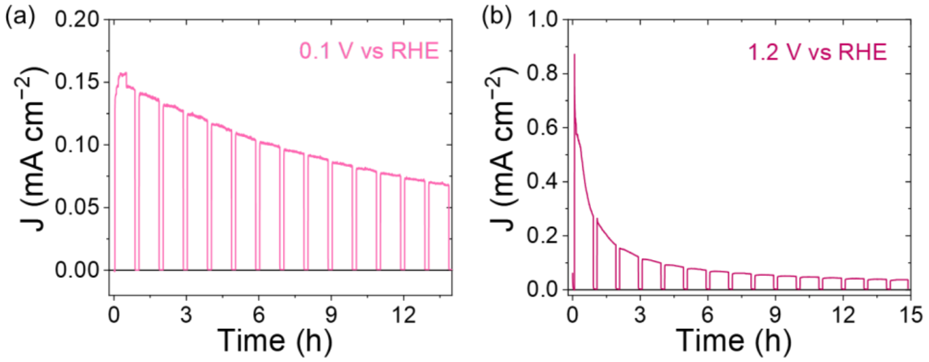

Figure S10. Chronoamperometry measurement of CN_x/ITO photoelectrodes under 0.5 sun simulated solar light and an external applied voltage of (a) 0.1 V and (b) 1.2 V vs RHE. Conditions: 1 compartment PEC cell (10 mL) in a 3-electrode configuration, 5 v/v % glycerol in 0.1 M Na_2SO_4 (pH 7), chopped light 50 min on/10 min off. All measurements were performed in triplicate.

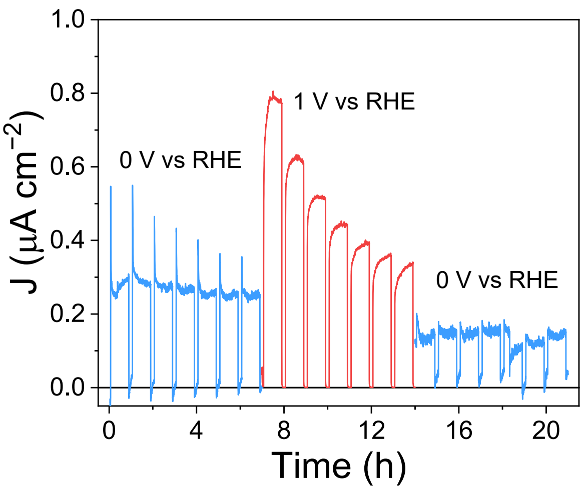

Figure S11. Chronoamperometry measurement of CN_x/ITO photoelectrodes under 3 suns simulated solar light and an external applied voltage of 0 V, 1 V, and 0 V vs RHE (7 h each). Conditions: 1 compartment PEC cell (10 mL) in a 3-electrode configuration, 5 v/v % glycerol in 0.1 M Na_2SO_4 (pH 7), chopped light 50 min on/10 min off. All measurements were performed in triplicate.

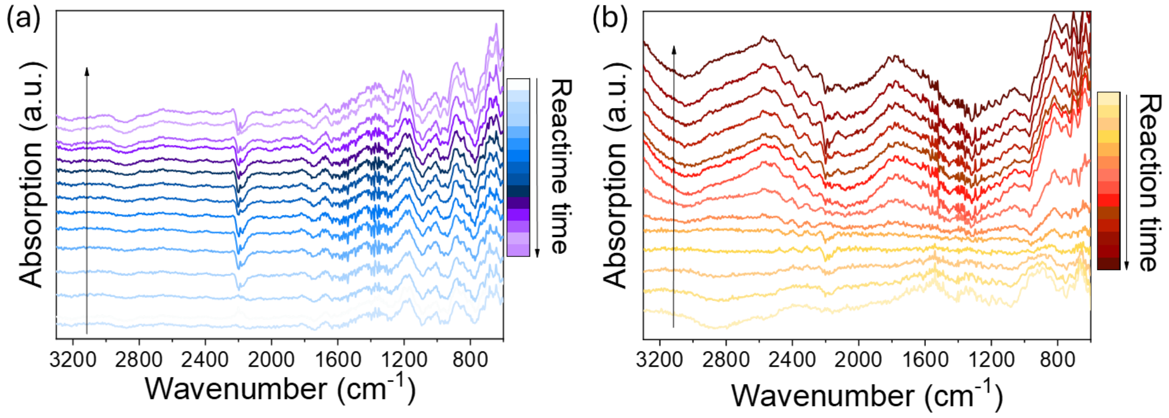

Figure S12. In-situ PEC IR analysis of CN_x/ITO photoanode as a function of applied potential. IR spectra of the CN_x/ITO photoanode under 1 sun illumination and (a) 0.2 V vs RHE or (b) 1.2 V vs RHE. Conditions: 3-electrode setup, Pt as counter electrode, Ag/AgCl as reference electrode, 5 v/v% glycerol in 0.1 M Na_2SO_4, customized 1-compartment PEC cell. Spectra were collected every 4 minutes over a total of 60 min.

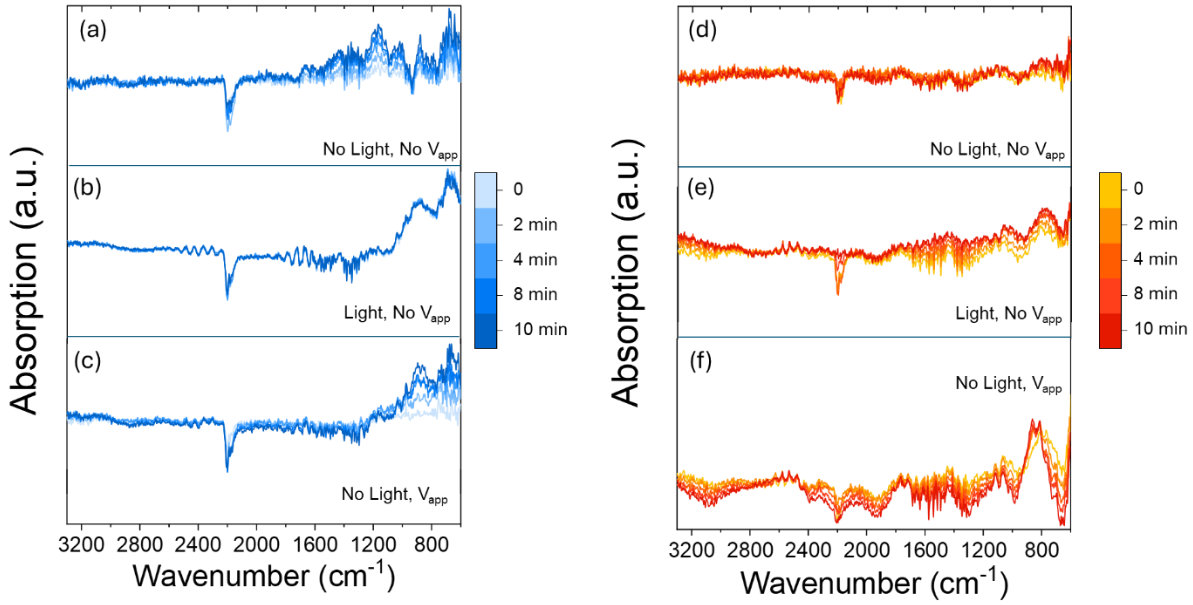

Figure S13. Control in-situ SR-FTIR spectra of the CN_x/ITO photoanode. Spectra were collected under 0.2 V (blue spectra, a to c) and 1.2 V vs RHE (red spectra, d to f). The IR control experiments were conducted under (a,d) no light and no external applied potential, under (b,e) 1 sun illumination and no potential, and (c,f) in the dark and applied potential. Each set of spectra was collected every 2 minutes over a total of 10 minutes. Conditions: 3-electrode setup, Pt as counter electrode, Ag/AgCl (saturated KCl) as reference electrode, 50 mM glycerol in 0.1 M Na_2SO_4, customized 1-compartment SR-FTIR-PEC cell. The photoelectrode active area was 0.05 cm^{-2}.

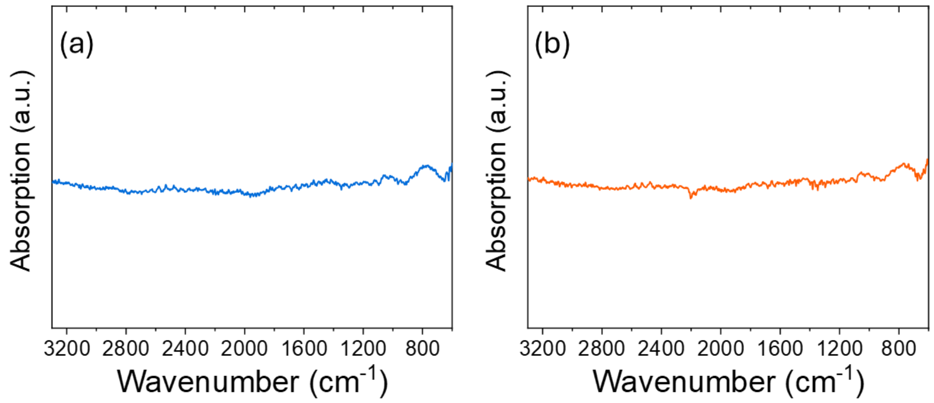

Figure S14. Baseline IR spectra for the of the CN_x/ITO photoanode tested under 1 sun illumination and (a) 0.2 V vs RHE or (b) 1.2 V vs RHE. Conditions: 3-electrode setup, Pt as counter electrode, Ag/AgCl as reference electrode, 5 v/v% glycerol in 0.1 M Na_2SO_4, customized 1-compartment PEC cell.

Extended Discussion of In-situ PEC IR in Figures S13 and S14. It is important to note that the IR spectra presented in this work are plotted in absorbance rather than transmittance, even though the presence of several negative signals might at first resemble transmittance data. In this context, the "bleached" peaks we refer to correspond to regions where the absorbance intensity decreases over time. More specifically, a bleach is defined as a reduction in the extinction coefficient of the vibrational mode, which implies that fewer absorbing species (or absorbing groups) contribute to that vibration as the reaction proceeds. Thus, a decreasing peak intensity in these absorbance spectra indicates a loss or transformation of the corresponding chemical functionality.

To ensure that any observed spectral change was related to the application of light and potential to the sample, IR spectra were first collected in the dark and under illumination with no external applied bias every 2 minutes, for a total of 10 minutes for both samples (**Figure S13**). From these control measurements, no change in absorbance was observed over time, confirming that the sample is stable in the electrolyte solution. Furthermore, the similarity between the spectra of the two samples ensures that any difference measured during PEC is not due to fundamental differences between samples. In general, the characteristic heptazine core vibration peaks between 1700 and 1000 cm^{-1} are still slightly visible over the broad absorption of the electrodes. The bleached peak, defined as a region with a lower extinction coefficient, at 2184 cm^{-1} was assigned to the characteristic C=N stretching of the cyanamide-functional group. The low density of functional groups compared to the main heptazine core and ITO nanoparticles can rationalize the bleached absorption pick. The consistency between light and dark measurements suggests that light alone does not affect the sample morphology or chemical nature, supporting the results obtained from PEC measurements described previously. The last control experiments consisted of subjecting the samples to either 0.2 V vs RHE (**Figure S13c**) or 1.2 V vs RHE (**Figure S13f**) in the dark. Under both conditions, new peaks are visible when compared to the unbiased tests, however, the sample held at 1.2 V vs RHE showed a small change in the relative peak intensity over time (10 min), not observed under 0.2 V vs RHE. More specifically, the new bleached pick at 3247 cm^{-1} grows in relative intensity, as for the absorbance bands at 1886 and 1236 cm^{-1}. Since the assignment of signals to specific chemical species of the carbon nitride material is non-trivial, the main apparent conclusion is that a more positive potential seems to affect the CN_x morphology, even in the dark. These preliminary results are consistent with results obtained from control electrochemical tests, which showed that the photoanode lost activity even in the dark under the same positive applied potential of 1.2 V vs RHE (**Figure S8**).

It is important to note that all spectra shown have the baseline already subtracted. The baseline was measured at the beginning of the measurements and are shown in **Figure S14**. However, the spectra shown in **Figure S12** were normalized to time zero spectra, not the baseline, of the measurement. In other words, t>4 min were normalized to the time zero spectra to provide insight into the dynamic surface transformations and stability of the carbon nitride photoanode under reaction conditions.

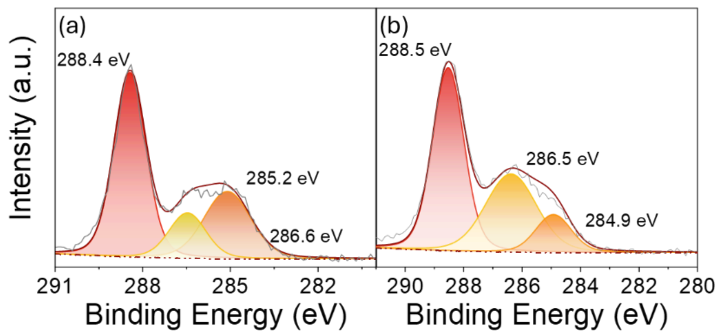

Figure S15. High-resolution XPS spectra of C 1s of the CN_x/ITO photoanode tested at 1 sun and (a) 0.1 V vs RHE and (b) 1.2 V vs RHE.

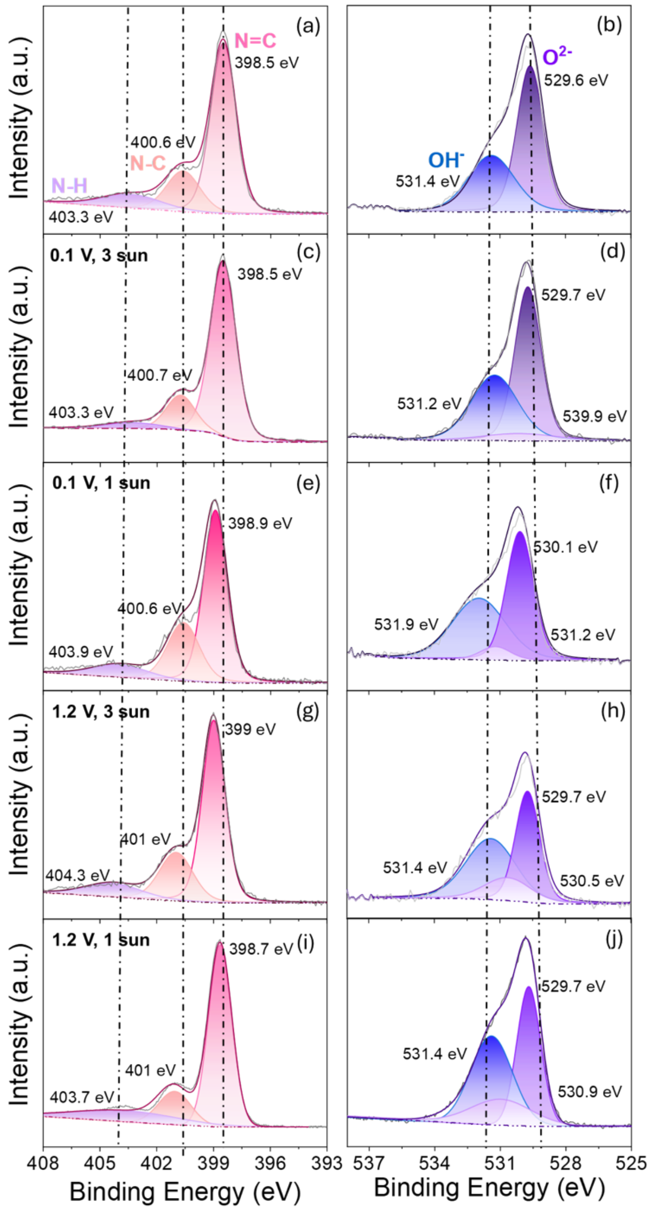

Figure S16. High-resolution XPS spectra of (a,c,e,g,i) N 1s, and (b,d,f,h,j) O 1s CN_x/ITO photoanode of the CN_x/ITO photoanodes unreacted, post 0.1 V vs RHE and 3 suns illumination, 0.1 V vs RHE and 1 sun illumination, and 1.2 V vs RHE and 1 sun illumination.

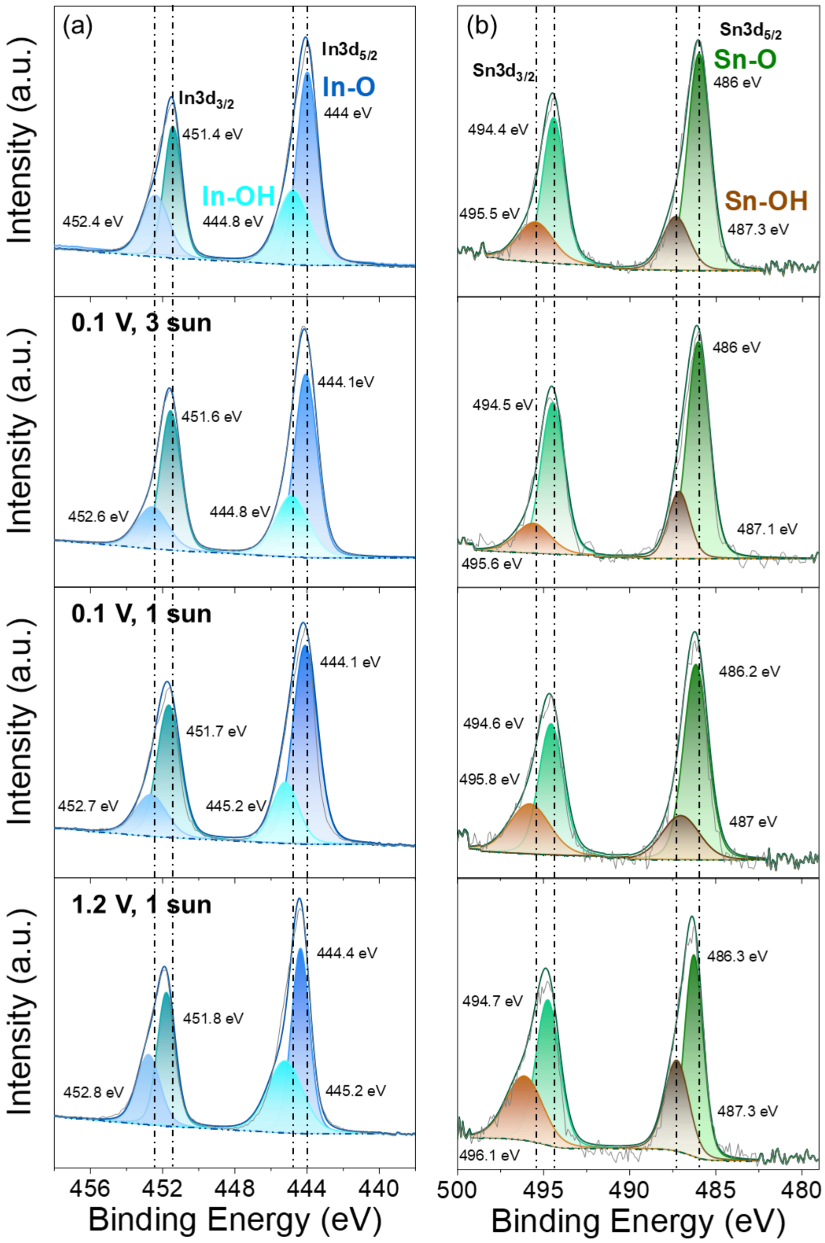

Figure S17. High-resolution XPS spectra of (a) In 3d and (b) Sn 3d of the CN_x/ITO photoanodes unreacted, post 0.1 V vs RHE and 3 suns illumination, 0.1 V vs RHE and 1 sun illumination, and 1.2 V vs RHE and 1 sun illumination.

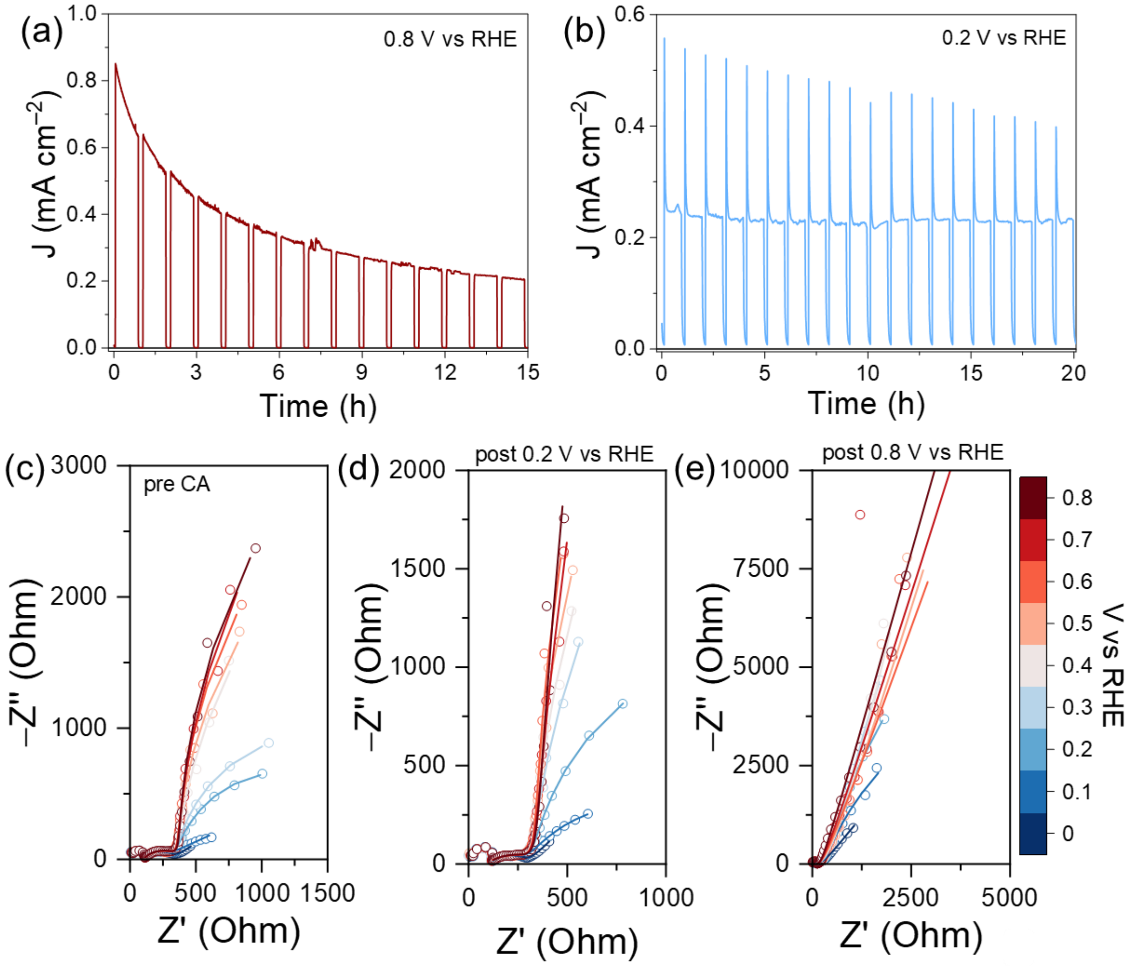

Figure S18. PEIS analysis of the effect of external applied potential on CN_x/ITO photoanodes stability at 3 sun. Chronoamperometries of CN_x/ITO under 3 sun simulated solar light (chopped, 50 min on/10 min off) at (a) 0.2 V vs RHE and (b) 0.8 V vs RHE. Voltage-dependent Nyquist plot measured by PEIS of CN_x/ITO photoanodes (c) before any CAs, and after holding the sample at (d) 0.2 V vs RHE, and (e) 0.8 V vs RHE, with corresponding fitting curves (solid lines). Conditions: 1-compartment PEC cell (10 mL) in a 3-electrode configuration, Pt mesh as counter electrode, Ag/AgCl as reference electrode, 5 v/v % glycerol in 0.1 M Na2SO4 (pH 7).

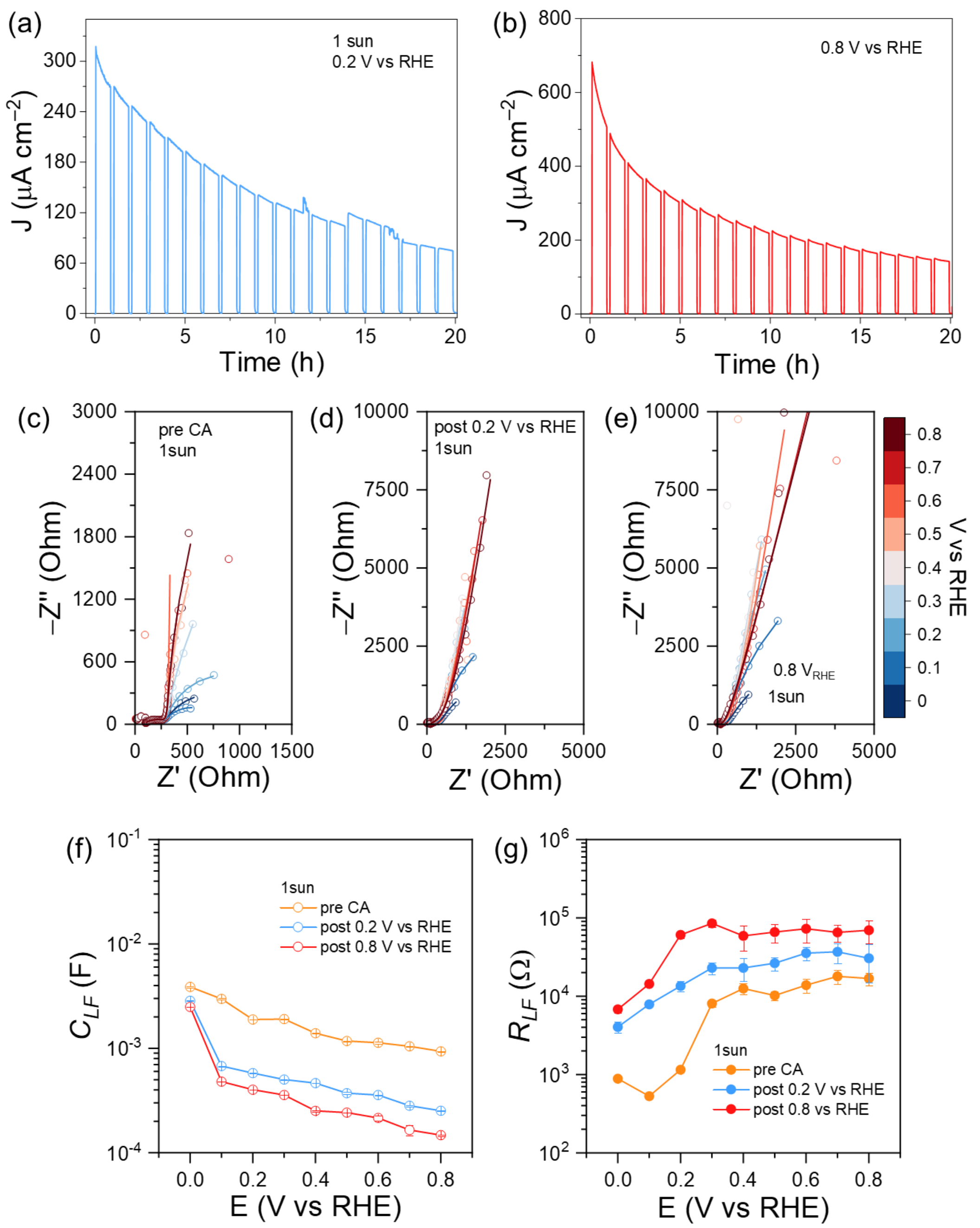

Figure S19. PEIS analysis of the effect of external applied potential on CN_x/ITO photoanodes stability at 1 sun. Chronoamperometries of CN_x/ITO under 1 sun simulated solar light (chopped, 50 min on/10 min off) at (a) 0.2 V vs RHE and (b) 0.8 V vs RHE. Voltage-dependent Nyquist plot measured by PEIS of CN_x/ITO photoanodes (c) before any CAs, and after holding the sample at (d) 0.2 V vs RHE, and (e) 0.8 V vs RHE, with corresponding fitting curves (solid lines). Voltage-dependent low-frequency (f) capacitance (C_{LF}) and (g) resistance (R_{LF}) values of CN_x/ITO photoanodes pre-CAs (yellow trace), post 0.2 V vs RHE tests (blue trace), and post 0.8 V vs RHE tests (red trace). Conditions: 1-compartment PEC cell (10 mL) in a 3-electrode configuration, Pt mesh as counter electrode, Ag/AgCl as reference electrode, 5 v/v % glycerol in 0.1 M Na2SO4 (pH 7).

Extended Discussion of Figures S18 and S19. As shown previously, even by testing the photoanodes at 0.1 V vs RHE or lower applied potentials, the photocurrent decays exponentially, even if less pronounced than the characteristic decay at higher voltages. The voltage-dependent Nyquist plots (from 0 to 0.8 V vs RHE) at 1 sun show the same two resistor-capacitor (RC) processes. The same equivalent two-RC circuit was employed to fit the data and derive the corresponding bulk capacitance and charge transfer resistance. However, whilst under high light intensities only the sample tested at 0.8 V vs RHE showed a sharp decrease in bulk capacitance, under 1 sun the external applied potential did not seem to have any effect in preventing the decline in C_{LF}. More specifically, the photoanode tested at 0.2 V vs RHE lost 71 ± 3 % of the initial capacitance and the one tested at 0.8 V vs RHE lost 82 ± 2 %. From these results, it is apparent that concentrated solar light is fundamental in ensuring long-term stability when subjecting the samples to low applied potentials, resulting in minor losses in the capability of the carbon nitride to hold charges over time.

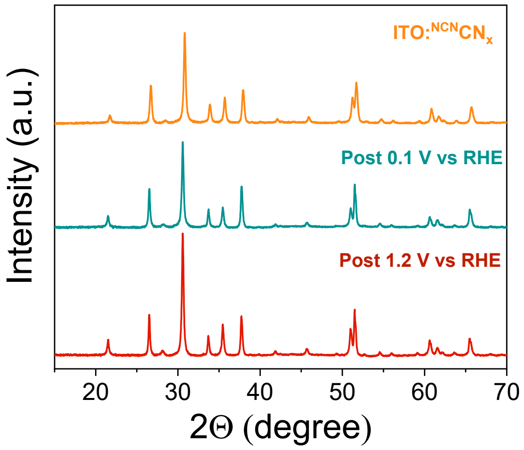

Figure S20. X-Ray diffraction (XRD) spectra of CN_x/ITO photoanodes before any CA tests and after testing the samples at 0.1 V and 1.2 V vs RHE under 3 suns illumination.

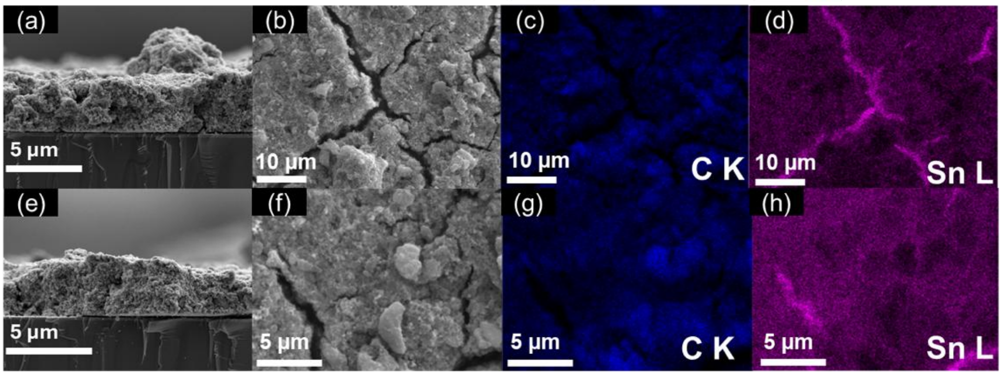

Figure S21. Post-CA SEM images of CN_x/ITO photoanode at 1.2 V vs RHE. (a,b) top-view SEM images of the CN_x/ITO photoelectrodes post 20-h CA at 0.1 V vs RHE and (e-f) 1.2 V vs RHE under 3 suns illumination. SEM EDX mapping showing (c,g) carbon (green) and (d,h) tin (pink) elemental distribution.

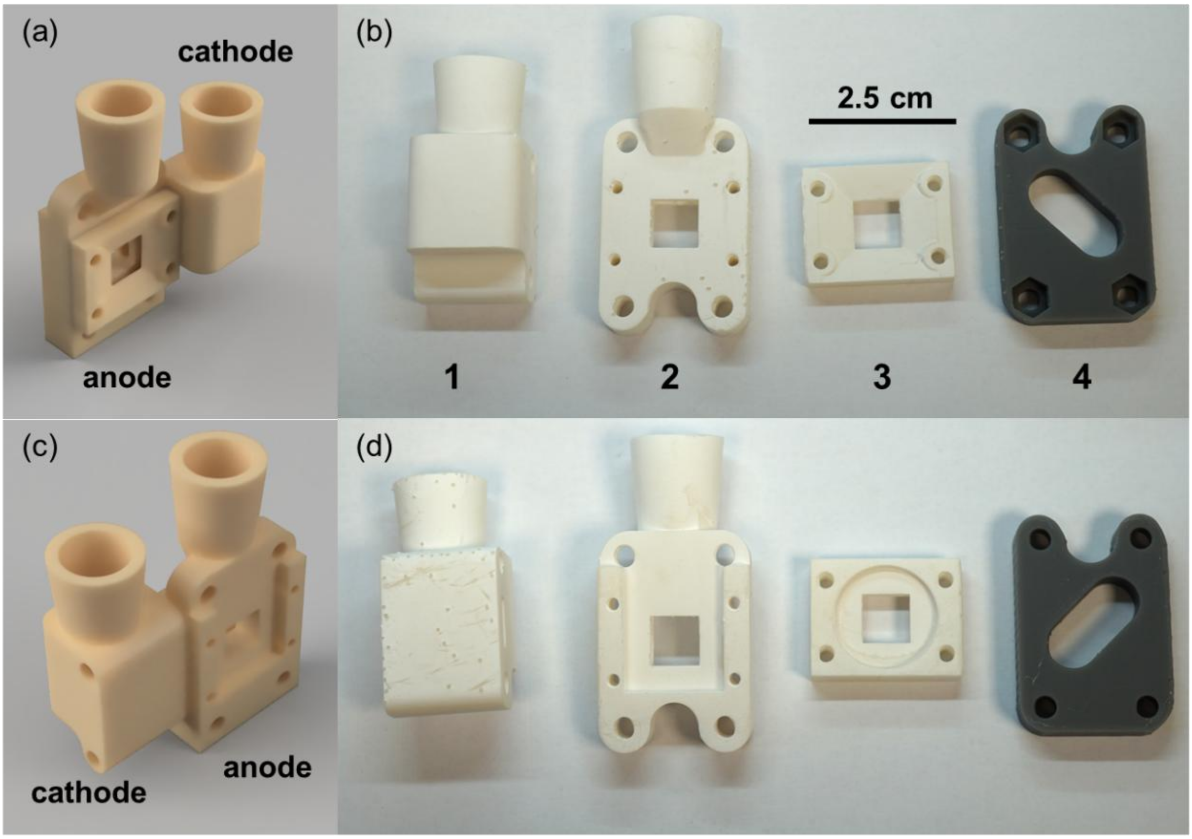

(**1**) Cathode compartment (**2**) Anode compartment (**3**) Front cover with quartz window

(**4**) Back cover

Figure S22. TEG-PEC cell design. (a) Front and (c) back view of the CAD rendering of the assembled TEG-PEC cell (Image of front view also shown in Figure 1a). Picture of the 3D-printed TEG-PEC pieces with (b) front and (d) back of the main cathode and anode pieces. The PEC cell parts were 3D printed with a Form 3B+ Low Force Stereolithography printer. The photoanode side was composed of 2 parts: (i) the front cover where the quartz window (1x1 cm^2) was positioned, and (ii) the main compartment with a reaction volume of 1.6 mL (total volume 2.5 mL) and a slot to position the carbon nitride electrode for front illumination. Accordingly, the TEG unit was placed between the back side of the photoanode and the aluminum wall of a cooling block (2.5 cm x 2.5 cm) kept at 21 ºC with a chiller, with both sides covered with thermal paste. The overall anode side was tightened using a back wall placed after the chilling unit, with holes to pass the chiller tubing connected to a main chiller unit set at 20 ºC. The cathode side was a unique piece with no transparent window and a reaction volume of only 2.6 mL.

Extended discussion of Figure S22. As shown in **Figure 1**, in the assembled TEG-PEC cell, the photoanode and cathode are placed one next to the other, separated by a bipolar membrane, facing the light source. It is fundamental to keep the catholyte and the anolyte separated to prevent product cross-over, which could poison the copper surface. Moreover, it is crucial to ensure that the carbon nitride material is not exposed to CO_2-reach atmosphere. More specifically, it has recently been proven that working under CO_2 atmosphere can accelerate the light-induced self-decomposition of graphitic carbon nitride, producing CO_2 and CO, which can ultimately affect reduction product quantification.[9] The use of a bipolar membrane ensured no product exchange between the two compartments, a fundamental condition not met when using a commercial Nafion® membrane in this specific condition, as shown in **Figure S23**.

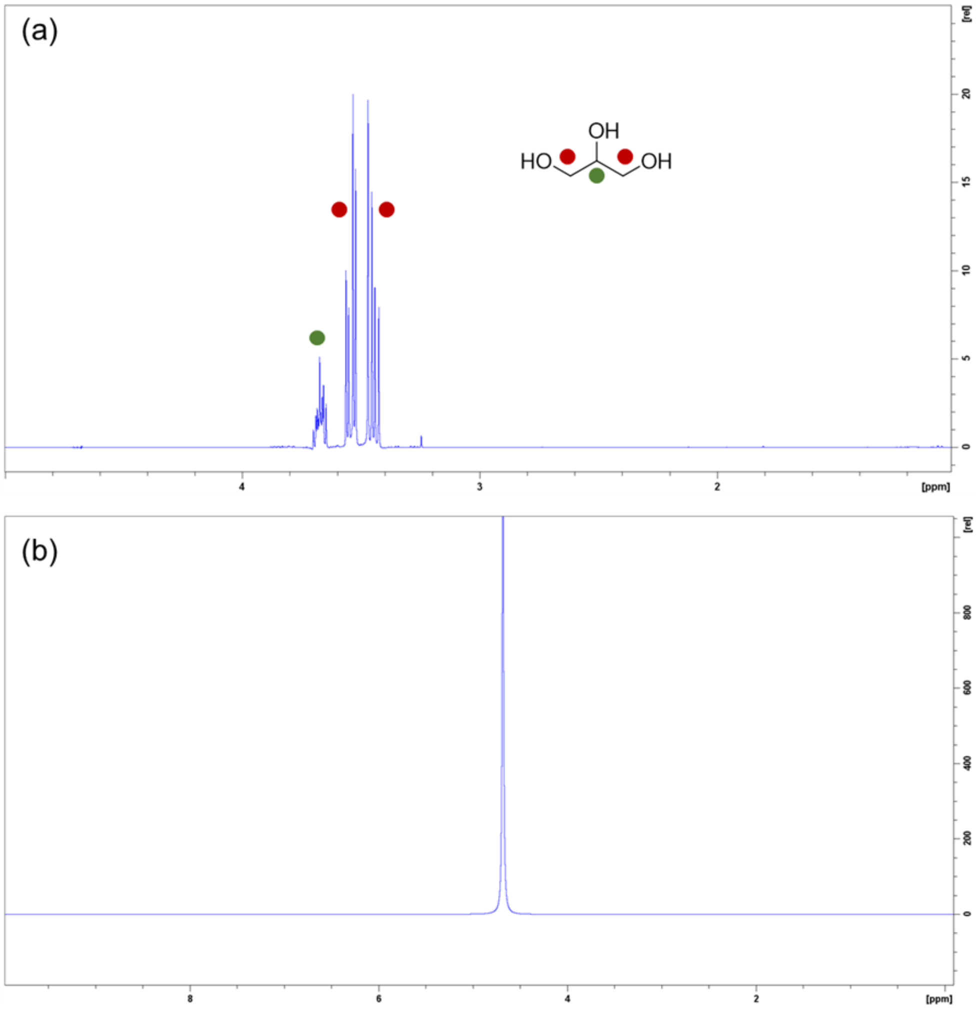

Figure S23. Analysis of catholyte solution post-CA with different membranes. ^{1}H NMR of catholyte solution after chronoamperometry with a (a) Nafion membrane and with a (b) bipolar membrane showing the presence and absence, respectively, of glycerol in the electrolyte due to cross-over. D_2O as solvent.

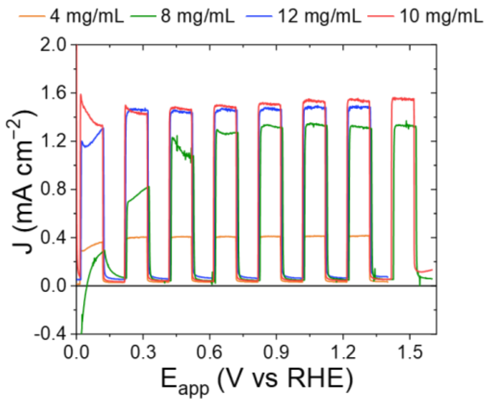

Figure S24. (a) Linear sweep voltammetry scans of spray-coated CN_x/ITO photoelectrodes (0.5 and 1 cm^2) prepared with different composite weight in 1 mL of ethanol. Conditions: 0.1 M aq. Na_2SO_4 (9 mL, pH 7), 4-MBA (50 mM), scan rate (10 mV s^{-1}), chopped simulated solar light (AM 1.5G, 100 mW cm^{-2}), N_2 atmosphere, room temperature. All measurements were performed in triplicate.

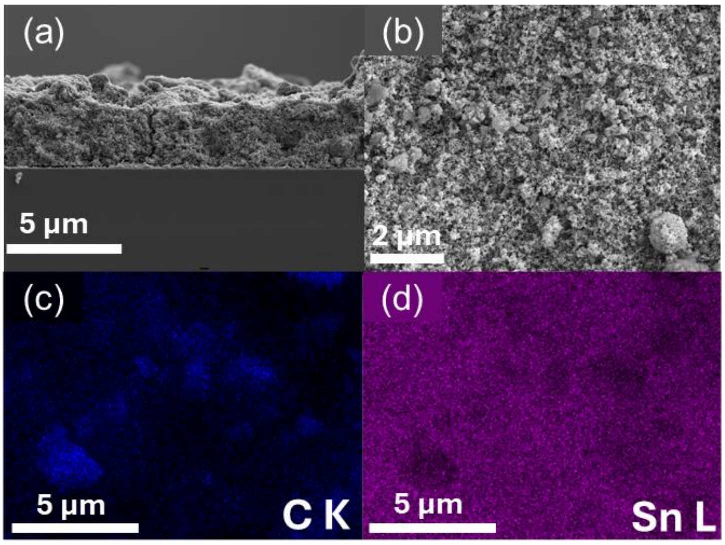

Figure S25. (a) Cross-section and (b) top-view SEM images of CN_x/ITO electrode (1 cm^2 active area; the same image is also shown in Figure 1d). Top-view SEM EDX mapping of CN_x:ITO photoanode showing the elemental distribution of (d) carbon and (e) tin. Samples were sputter-coated with a 10 nm layer of Cr prior to measurement.

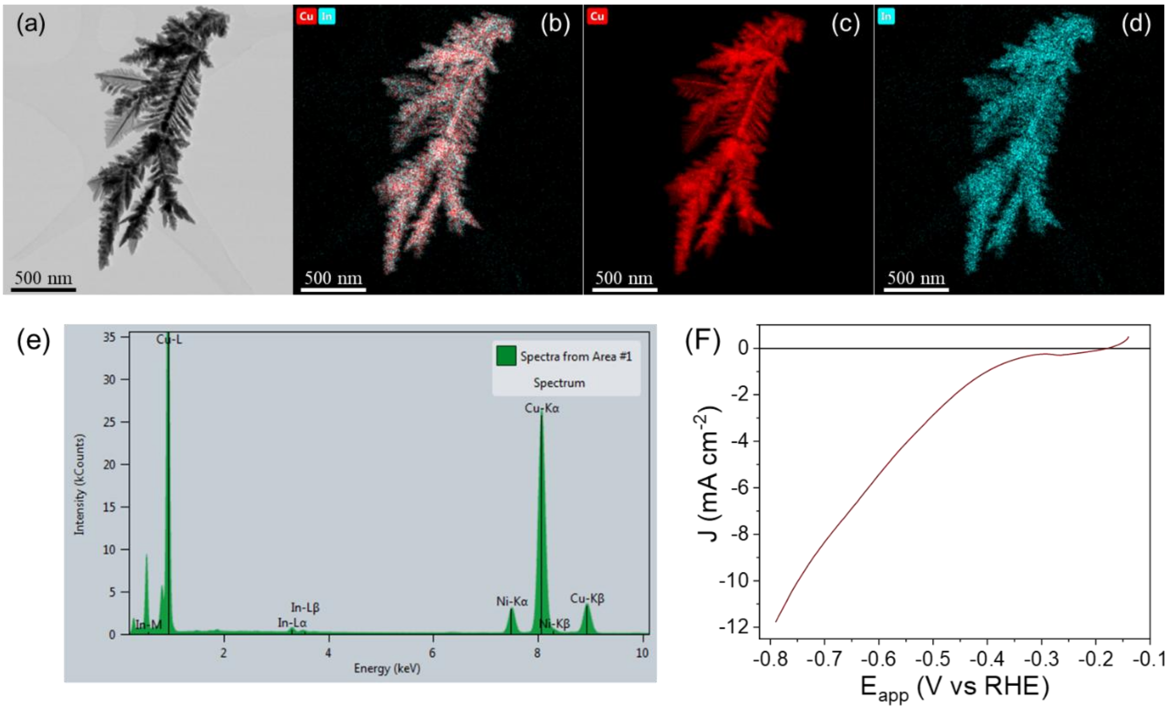

Figure S26. $Cu_{97}In_3$ characterization. (a) High-resolution transmission electron microscopy (HR-TEM) of Cu97In3 dendrite. EDX mapping of (b) Cu97In3 showing the uniform distribution of (c) copper (red) and (d) indium (light blue) elemental distribution (the same image is also shown in Figure 1c). (e) Energy-dispersive X−ray spectroscopy (EDX) mapping element distribution in a CuIn alloy dendrite. (f) Linear sweep voltammetry scan of $Cu_{97}In_3$ electrode. Conditions: 3-electrode configuration with Pt as counter and Ag/AgCl as reference electrodes in a CO_2-saturated (pH 7.2) 0.5 M aqueous $KHCO_3$ electrolyte solution, 20 mV s^{-1}.

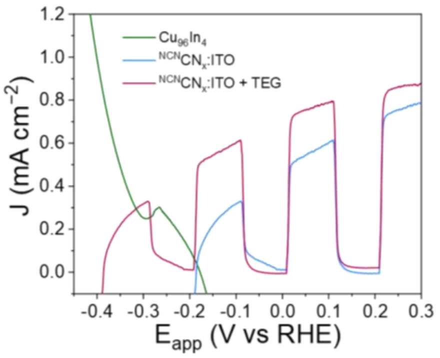

Figure S27. Current crossover plots for 3-suns irradiation on CN_x/ITO photoanode (blue) and CN_x/ITO|TEG (red) with the $Cu_{97}In_3$ cathode (green, currents inverted to show current intercept with photoanode) acquired in a 3-electrode setup.

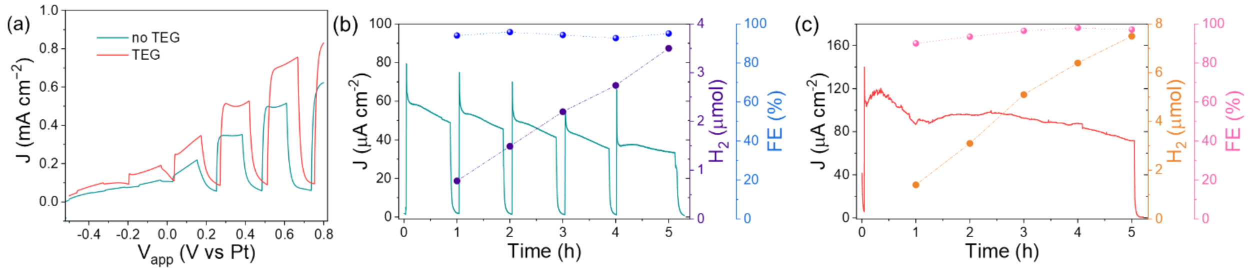

Figure S28. TEG-PEC cell first long-term test. (a) LSV scans of the final TEG|CN_x/ITO PEC setup under 3 suns illumination without (green trace) and with (red trace) the TEG unit wired to the photoanode, with Pt as counter electrode. CA of the TEG|ITO/CN_x PEC cell over 5 h chopped and constant light (b) without and (c) with the TEG unit wired to the photoanode, with the amount of H_2 produced and FE over time. Conditions: anolyte solution consisted of 1.5 mL of 5 v/v% glycerol in aqueous 0.1 M Na_2SO_4, purged with N_2 for 15 min; catholyte solution consisted of 2.5 mL of 0.5 M $KHCO_3$, purged with CO_2 for 15 min, 1 cm^2 photoanode area.

Extended Discussion of Figure S28. The TEG-PEC cell was troubleshooted first with Pt as counter electrode for H_2 production. The photoanode compartment was filled with 1.5 mL of aqueous 5 v/v% glycerol 0.1 M Na_2SO_4 solution and purged with N_2 for 30 min, whilst the cathode side was filled with 2.5 mL of aqueous 0.5 M $NaHCO_3$ and purged with CO_2 for 30 min, to be consistent with the conditions that will be employed with the CO_2-reduction electrocatalyst. The H_2 evolved was measured over time by sampling the catholyte headspace every hour. LSV and five-hour-long CAs tests were performed under 3 sun concentrated solar light without and with the TEG unit wired to the CN_x/ITO photoanode (1 cm^2 area). The thermoelectric unit under concentrated irradiation (3 sun) allowed for shifting the onset potential of ~200 mV after only 10 minutes of constant illumination and doubling the photocurrent in the unbiased PEC device. In all tests, the faradaic efficiency stayed over 90 % for oxidation and reduction products. In the anolyte, the same product selectivity measured in 3-electrode 1-compartment CAs was observed, with a ratio of 2:1 of glyceraldehyde and dihydroxyacetone.

The thermoelectric generator (TEG) integrated in this PEC device operates on the Seebeck effect, whereby a temperature difference (ΔT) across a thermoelectric material generates an electromotive force. When one side of the TEG is heated (in our case, by the unused wavelengths from the

photoanode and IR radiation), and the opposite side is maintained at a lower temperature (via a chiller plate), charge carriers within the thermoelectric legs (electrons in n-type, holes in p-type) diffuse from the hot side to the cold side. This carrier diffusion produces a (thermos)voltage given by:

$$V_{TEG} = S \times \Delta T$$

where S is the Seebeck coefficient (V K^{-1}) of the thermoelectric material, and ΔT is the temperature gradient. For the commercial Adaptive Peltier Module (21.2W, 3.9A, 8.8V, 20 x 20mm) S typically ranges from 150–250 μV K^{-1} per leg pair, allowing a ΔT of ~10–15 °C to produce ~0.15–0.30 V in a single module, as confirmed by the 200 mV shift measured with a difference of 15 °C.

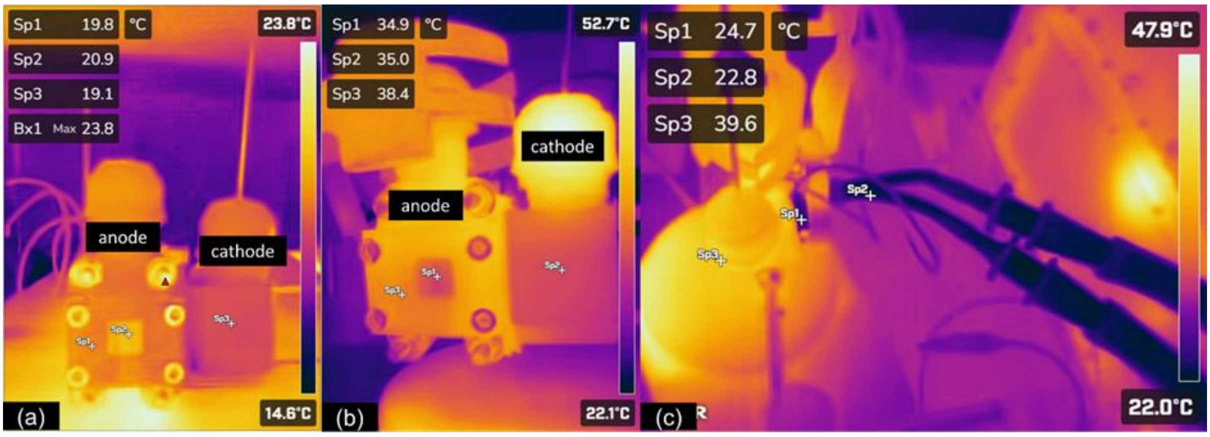

Figure S29. Infrared thermal images of the TEG-PEC cell. Front view infrared thermal images of the TEG-PEC cell (a) before and (b) after CA measurement under 3 suns-concentrated solar light. (c) Lateral view infrared thermal image of the TEG-PEC setup after CA measurement under 3 suns concentrated solar light. The thermal images were collected with a FLIR ONE Pro-Series camera, with a sensitivity that detects temperature differences down to 70 mK.

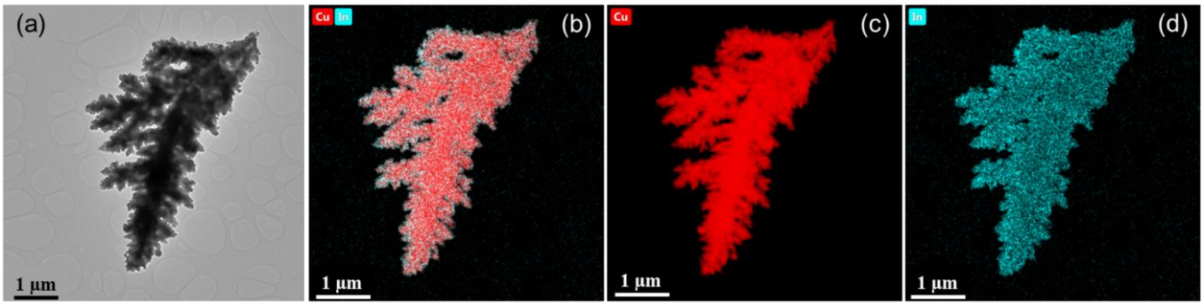

Figure S30. Post-catalysis TEM EDX analysis of Cu-based cathode. (a) High-resolution transmission electron microscopy (HR-TEM) of Cu-based cathode post 70 h CA, now a $Cu_{99}In_1$ distribution. EDX mapping of (b) $Cu_{99}In_3$ showing the uniform distribution of (c) copper (red) and (d) indium (light blue) elemental distribution.

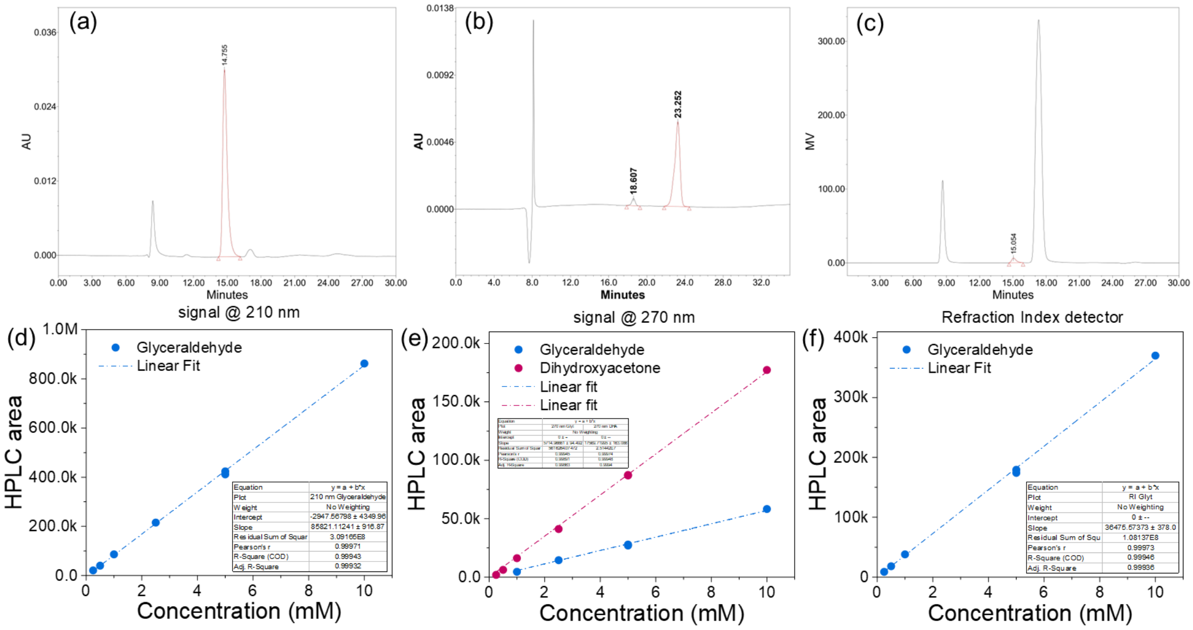

Figure S31. HPLC calibration curve and analysis for glycerol oxidation product analysis. HPLC spectrum of a calibration mixture of glyceraldehyde (15 min retention time) and dihydroxyacetone (23 min retention time) at (a) 210 nm, (b) 270 nm, and (c) RI signal, with (d-f) corresponding calibration curve calculations.

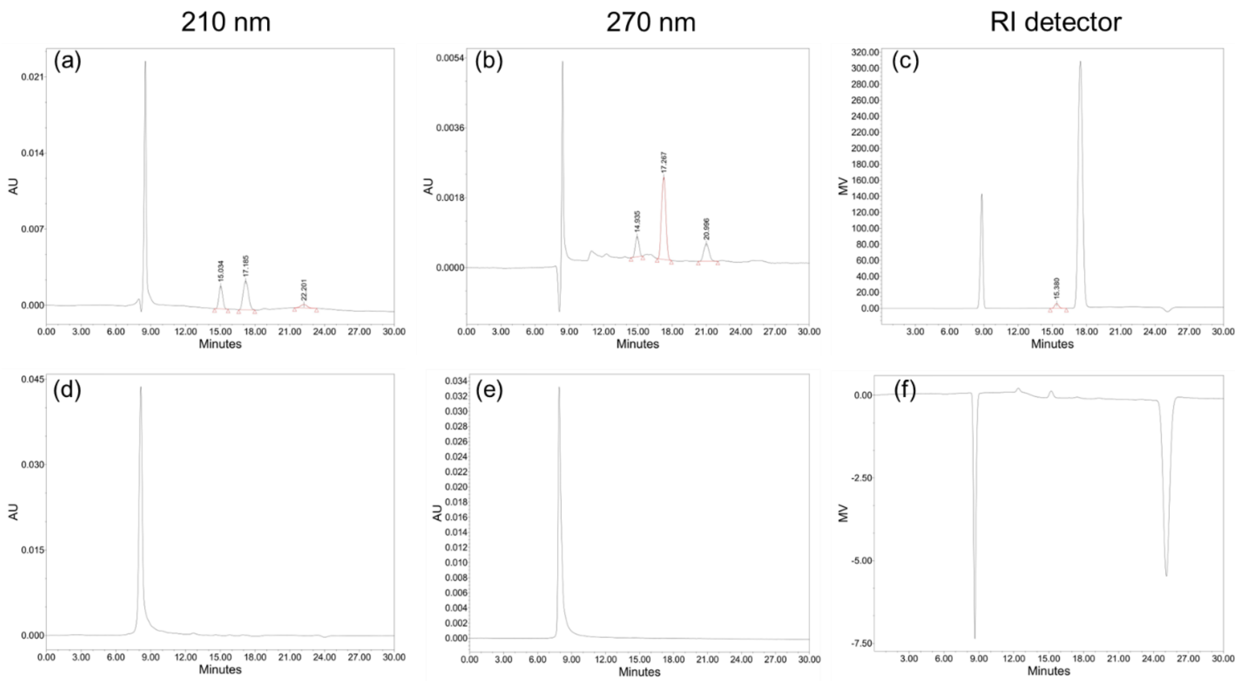

Figure S32. HPLC glycerol oxidation product analysis. HPLC spectrum of anolyte at (a) 210 nm, (b) 270 nm, (c) RI detector and the corresponding (d-f) catholyte response at that same wavelength after 70 h chopped illumination under 3 suns illumination.

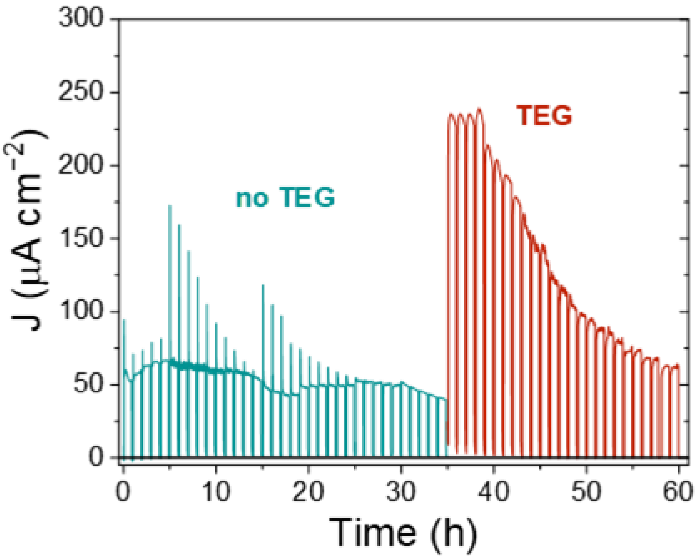

Figure S33. Duplicate of chronoamperometry test of the CN_x/ITO|TEG||$Cu_{97}In_3$ device over 60 h chopped light without and with the TEG unit wired to the system. Conditions: 3 sun-simulated solar light, two-electrode configuration, two-compartment PEC-TEG cell, 5 v/v % glycerol 0.1 M Na_2SO_4 as anolyte (1.5 mL) purged for 15 min with N_2, and 0.5 M $NaHCO_3$ as catholyte (2.5 mL) purged for 15 min with CO_2, 1 cm^2 photoanode area.

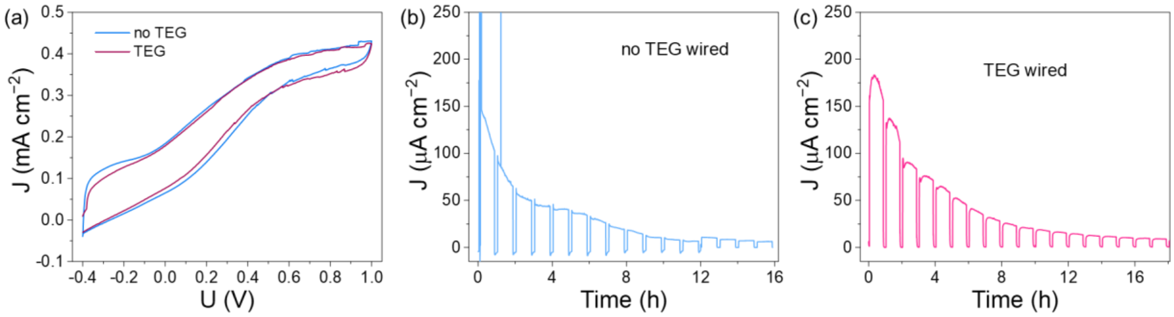

Figure S34. TEG-PEC cell performance evaluation under 1 sun illumination. (a) Reverse CV scans of the assembled TEG|ITO/CN_x||$Cu_{97}In_3$ cell under 1 sun illumination, with and without the TEG unit wired to the photoanode. CA measurements of the assembled setup (b) without and (c) with the TEG unit wired, showing exponential photocurrent decay over time. Conditions: anolyte solution consisted of 1.5 mL of 5 v/v% glycerol in aqueous 0.1 M Na_2SO_4, purged with N_2 for 15 min; catholyte solution consisted of 2.5 mL of 0.5 M $KHCO_3$, purged with CO_2 for 15 min, 1 cm^2 photoanode area., 1 sun simulated solar light.

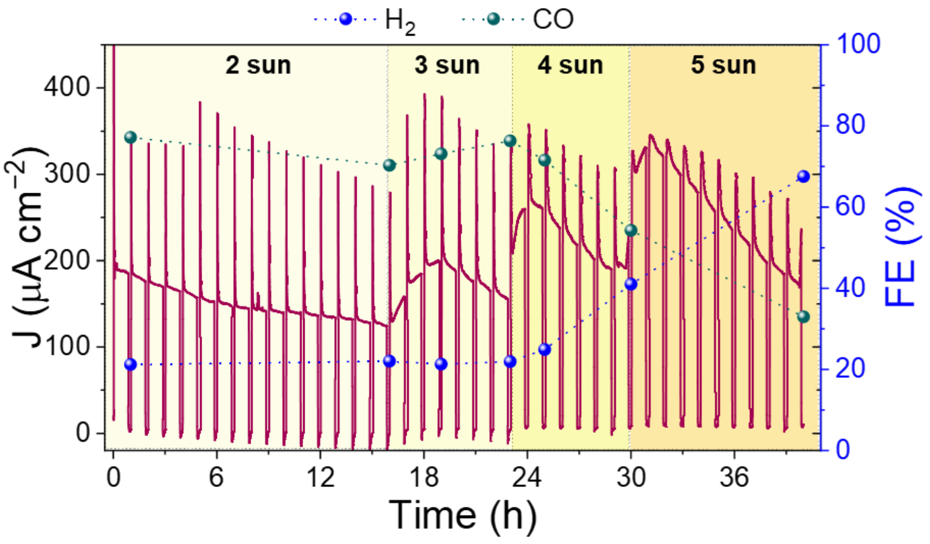

Figure S35. Light concentration effect on CN_x/ITO|TEG||$Cu_{97}In_3$ device product selectivity. CA of the CN_x/ITO|TEG||$Cu_{97}In_3$ device over 41 h chopped light under increasing light intensities, with H_2 and CO FE measured over time. Conditions: TEG unit wired to the device, two-electrode configuration, two-compartment PEC-TEG cell, 5 v/v % glycerol 0.1 M Na_2SO_4 as anolyte (1.5 mL), 0.5 M $NaHCO_3$ as catholyte (2.5 mL). The temperature of the anolyte reached 50ºC at 5 suns.

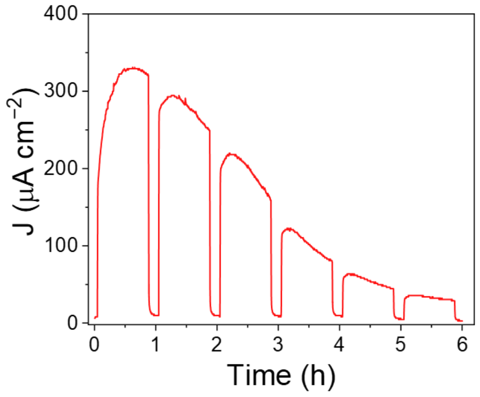

Figure S36. Chronoamperometry test of CN_x/ITO photoanode (1 cm^2 area) under 6 suns-simulated solar light in a 2-electrode configuration in a 2-compartment PEC cell with Pt as counter electrode. Conditions: anolyte solution consisted of 5 v/v% glycerol in aqueous 0.1 M Na_2SO_4, purged with N_2 for 15 min; catholyte solution consisted of 0.5 M $NaHCO_3$, purged with CO_2 for 15 min. All measurements were performed in triplicate.

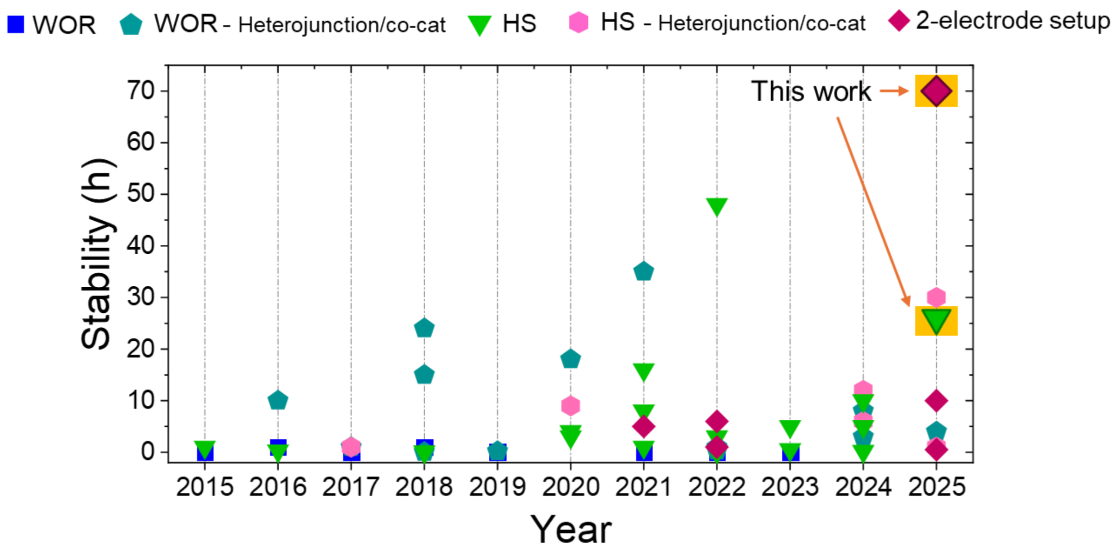

Figure S37. Comparison with representative carbon nitride-based photoanodes. Comparison of the stability (defined as the testing time retaining >50 % of the initial photocurrent), expressed in hours, of our CN_x/ITO photoanodes in a 3-electrode setup (highlighted larger green triangle) and as part of our CN_x/ITO|TEG||$Cu_{97}In_3$ device (highlighted larger dark red rhombus) with other selected representative carbon nitride-based photoanodes reported over the paste decade for both WOR and hole scavenger (HS) oxidation, with or without the addition of a co-catalyst or the formation of a heterojunction. Our single-light absorber CN_x-based PEC device (under zero applied voltage for glycerol valorization) is over 7 times more stable than any previously reported CN_x-driven PEC device, while in a 3-electrode configuration, our unmodified single-absorber photoanode ranks as the second-most stable ever reported (see **Table S2** for further details, corresponding references, and abbreviations).

Supporting Tables

Table S1. Exclusion control experiments of CN_x/ITO|TEG||$Cu_{97}In_3$ device.

Test	TEG	Light	$^{NCN}CN_x$	$Cu_{97}In_3$	H_2	CO	Glyt	DHA
**1**	NO	NO	YES	YES	n.d	n.d	n.d	n.d
**2**	NO	YES	YES	NO	n.d	n.d	traces	n.d
**3**	NO	YES	NO	NO	n.d	n.d	n.d	n.d
**4**	NO	NO	NO	NO	n.d	n.d	n.d	n.d

Table S2. Comparison with representative CN_x-based photoanodes. Comparison of the stability (defined as the testing time retaining >50 % of the initial photocurrent), expressed in hours, of our CN_x/ITO photoanodes in a 3-electrode setup (second-to-last entry) and as part of our CN_x/ITO|TEG||$Cu_{97}In_3$ device (last entry) with other selected representative carbon nitride-based photoanodes reported over the paste decade. Where no co-catalyst or heterojunction was employed, and the photoanode was based simply on a CN_x material (either synthetically modified or with the use of a conductive binder) no further details are given herein. 'WOR' indicates "water oxidation reaction, 'HS' indicates a generic "hole scavenger", 'TEOA' indicates "triethanolamine", '4-MBA' indicates "4-methylbenzyl alcohol, 'BnOH' indicates "benzyl alcohols, 'NRGO' indicates "nitrogen-doped reduced graphene", 'Co-Pi' indicates "cobalt phosphate-based co-catalyst".

#	Year	Stability	Photocurrent (mA cm^{-2})	Oxidation substrate	Applied potential	Setup	Photoanode	Ref
1	2015	60 min	0.12	Na_2S, ethanol	1.23 V vs RHE	3-electrode	-	[10]
2	2015	5 min	0.04	WOR	1.23 V vs RHE	3-electrode	-	[10]
3	2016	10 h	0.15	WOR	1.23 V vs RHE	3-electrode	CN_x/NRGO/NiFe	[11]
4	2016	16 min	0.125	TEOA	1.23 V vs RHE	3-electrode	-	[12]
5	2016	60 min	0.06	Na_2SO_3, Na_2S	1.23 V vs RHE	3-electrode	-	[13]
6	2017	35 s	0.063	WOR	1.23 V vs RHE	3-electrode	-	[14]
7	2017	60 min	0.35	Na_2SO_3	1.23 V vs RHE	3-electrode	Heterojunction g-C_3N_4/$BiVO_4$	[15]
8	2017	60 min	0.05	WOR	1.23 V vs RHE	3-electrode	Protective layer g-CN-TiO_2	[16]
9	2018	24 h	0.6	WO	1.23 V vs RHE	3-electrode	Fe_2O_3/g-CN_x/Co-Pi	[17]
10	2018	5 min	0.66	TEOA	1.23 V vs RHE	3-electrode	-	[18]
11	2018	3 min	0.72	WO	1.23 V vs RHE	3-electrode	TiO_2/g-CN_x	[19]
12	2018	15 h	0.25	WOR	1.23 V vs RHE	3-electrode	TiO_2/g-CN_x	[20]
13	2018	1 h	0.125	WOR	1.23 V vs RHE	3-electrode		[21]
14	2019	2 min	0.15	WOR	1.23 V vs RHE	3-electrode	-	[22]
15	2019	5 min	0.03	WOR	1.23 V vs RHE	3-electrode	-	[23]
16	2019	12 min	0.08	WOR	1.23 V vs RHE	3-electrode	TiO_2/Pd-gCN_x/$Ni(OH)_2$	[24]
17	2020	4 h	0.12	Na_2S	1.23 V vs RHE	3-electrode	-	[25]
18	2020	9 h	0.35	TEOA	1.23 V vs RHE	3-electrode	Type-II heterojunction	[26]
19	2020	18 h	0.2	WOR	1.23 V vs RHE	3-electrode	Type-II heterojunction	[27]
20	2020	3 h	0.1	TEOA	1.23 V vs RHE	3-electrode	-	[28]
21	2021	16 h	0.06	TEOA	1.23 V vs RHE	3-electrode	-	[29]
22	2021	5 min	0.1	WOR	1.23 V vs RHE	3-electrode	-	[29]
23	2021	8 h	0.1	4-MBA	1.1 V vs RHE	3-electrode	-	8
24	2021	5 h	0.15	MeOH	0.5 V vs Pt	2-electrode	-	8
25	2021	35 h	0.41	WOR	1.23 V vs RHE	3-electrode	CN_x/$NiFeO_xH_y$	[30]
26	2021	60 min	0.3	WOR	1.23 V vs RHE	3-electrode	-	[31]
27	2021	2 min	0.4	TEOA	1.23 V vs RHE	3-electrode	-	[31]

28	2022	60 min	0.009	WOR	unbiased	2-electrode	-	[32]
29	2022	6 h	0.02	MeOH	unbiased	2-electrode	-	[32]
30	2022	7 min	0.06	WOR	1.23 V vs RHE	3-electrode	-	[33]
31	2022	7 min	0.16	TEOA	1.23 V vs RHE	3-electrode	-	[33]
32	2022	20 min	0.2	WOR	1.23 V vs RHE	3-electrode	CoS_2-CN_x	[34]
33	2022	48 h	0.3	BnOH	1.23 V vs RHE	3-electrode	CN_x modified with reduced graphene oxide	[35]
34	2022	3 h	1.2	4-MBA	1.23 V vs RHE	3-electrode	-	3
35	2022	1 h	0.15	WOR	1.12 V vs RHE	3-electrode	CN_x/TiO_2/Co-polyoxometalate	[36]
36	2023	5 h	0.3	TEOA	1.23 V vs RHE	3-electrode	-	[37]
37	2023	30 min	1.2	TEOA	1.23 V vs RHE	3-electrode	B- and Na-doped CN_x	[38]
38	2023	4 min	0.65	Na_2SO_3, Na_2S	1.23 V vs RHE	3-electrode	-	[39]
39	2024	8 h	0.16	WOR	1.23 V vs RHE	3-electrode	CN_x/ZnSe heterojunction	[40]
40	2024	12 h	0.32	TEOA	1.23 V vs RHE	3-electrode	CN_x/ZnSe heterojunction	[40]
41	2024	5 h	0.2	TEOA	1.23 V vs RHE	3-electrode	-	[41]
42	2024	6 h	0.1	WO	1.23 V vs RHE	3-electrode	CN_x-Ru-based co-cat	[42]
43	2024	10 min	0.18	TEOA	1.23 V vs RHE	3-electrode	-	[43]
44	2024	10 h	0.2	TEOA	1.23 V vs RHE	3-electrode	-	[44]
45	2024	3 h	0.6	WOR	1.23 V vs RHE	3-electrode	CN_x/TiO_2	[45]
46	2025	1 h	0.44	TEOA	1.23 V vs RHE	3-electrode	CN_x/Ni-Co-based co-cat	[46]
47	2025	30 min	0.012	WOR	Unbiased	2-electrode	-	[46]
48	2025	10 h	0.08	4-MBA	Unbiased	2-electrode	-	[47]
49	2025	4 h	0.3	WOR	1.23 V vs RHE	3-electrode	Yttrium-CN_x	[48]
50	2025	30 h	0.4	TEOA	1.23 V vs RHE	3-electrode	Yttrium-CN_x	[48]
51	**2025**	**26 h**	**0.32**	**glycerol**	**1.23 V vs RHE**	**3-electrode**	**-**	**This work**
52	**2025**	**70 h**	**0.05 to 0.2**	**glycerol**	**Unbiased**	**2-electrode**	**-**	**This work**

Supporting References

1. Kuriki, R. & Maeda, K. Development of hybrid photocatalysts constructed with a metal complex and graphitic carbon nitride for visible-light-driven CO_2 reduction. *Phys. Chem. Chem. Phys.* **19**, 4938–4950 (2017).

2. Kuriki, R. *et al.* Robust Binding between Carbon Nitride Nanosheets and a Binuclear Ruthenium(II) Complex Enabling Durable, Selective CO_2 Reduction under Visible Light in Aqueous Solution. *Angew. Chem. Int. Ed.* **56**, 4867–4871 (2017).

3. Pulignani, C. *et al.* Rational Design of Carbon Nitride Photoelectrodes with High Activity Toward Organic Oxidations. *Angew. Chem. Int. Ed.* **61**, e202211587 (2022).

4. Rahaman, M. *et al.* Selective CO production from aqueous CO_2 using a $Cu_{96}In_4$ catalyst and its integration into a bias-free solar perovskite-$BiVO_4$ tandem device. *Energy Environ. Sci.* **13**, 3536–3543 (2020).

5. Trasatti, S. The absolute electrode potential: An explanatory note (Recommendations 1986). *Pure Appl. Chem.* **58**, 955–966 (1986).

6. Bard, A. J. & Faulkner, L. R. *Electrochemical Methods: Fundamentals and Applications*. (Wiley, 2000).

7. Han, C. *et al.* Nanostructured hybrid catalysts empower the artificial leaf for solar-driven ammonia production from nitrate. *Energy Environ. Sci.* **17**, 5653–5665 (2024).

8. Adler, C. *et al.* Sol−Gel Processing of Water-Soluble Carbon Nitride Enables High-Performance Photoanodes. *ChemSusChem* **14**, 2170–2179 (2021).

9. Chen, P. *et al.* Rapid Self-Decomposition of g-C_3N_4 During Gas-Solid Photocatalytic CO_2 Reduction and Its Effects on Performance Assessment. *ACS Catal.* **12**, 4560–4570 (2022).

10. Bian, J. *et al.* Thermal vapor condensation of uniform graphitic carbon nitride films with remarkable photocurrent density for photoelectrochemical applications. *Nano Energy* **15**, 353–361 (2015).

11. Hou, Y., Wen, Z., Cui, S., Feng, X. & Chen, J. Strongly Coupled Ternary Hybrid Aerogels of N-deficient Porous Graphitic-C_3N_4 Nanosheets/N-Doped Graphene/NiFe-Layered Double Hydroxide for Solar-Driven Photoelectrochemical Water Oxidation. *Nano Lett.* **16**, 2268–2277 (2016).

12. Xu, J. & Shalom, M. Electrophoretic Deposition of Carbon Nitride Layers for Photoelectrochemical Applications. *ACS Appl. Mater. Interfaces* **8**, 13058–13063 (2016).

13. Bian, J. *et al.* Efficiency Enhancement of Carbon Nitride Photoelectrochemical Cells via Tailored Monomers Design. *Adv. Energy Mater.* **6**, 4–9 (2016).

14. Lv, X., Cao, M., Shi, W., Wang, M. & Shen, Y. A new strategy of preparing uniform graphitic carbon nitride films for photoelectrochemical application. *Carbon* **117**, 343–350 (2017).

15. Wang, Y., Sun, J., Li, J. & Zhao, X. Electrospinning Preparation of Nanostructured g-C_3N_4/$BiVO_4$ Composite Films with an Enhanced Photoelectrochemical Performance. *Langmuir* **33**, 4694–4701 (2017).

16. Bian, J. *et al.* C=C π Bond Modified Graphitic Carbon Nitride Films for Enhanced Photoelectrochemical Cell Performance. *Chem. Asian J.* **12**, 1005–1012 (2017).

17. An, X., Hu, C., Lan, H., Liu, H. & Qu, J. Strongly Coupled Metal Oxide/Reassembled Carbon Nitride/Co-Pi Heterostructures for Efficient Photoelectrochemical Water Splitting. *ACS Appl. Mater. Interfaces* **10**, 6424–6432 (2018).

18. Peng, G., Volokh, M., Tzadikov, J., Sun, J. & Shalom, M. Carbon Nitride/Reduced Graphene Oxide Film with Enhanced Electron Diffusion Length: An Efficient Photo-Electrochemical Cell for Hydrogen Generation. *Adv. Energy Mater.* **8**, 1800566 (2018).

19. Wang, R. *et al.* Unconventional gas-based bottom-up, meter-area-scale fabrication of hydrogen-bond free g-CN nanorod arrays and coupling layers with TiO2 toward high-efficiency photoelectrochemical performance. *Nanoscale* **10**, 3342–3349 (2018).

20. Kang, S., Jang, J., Pawar, R. C., Ahn, S. & Lee, C. S. Direct coating of a g-C_3N_4 layer onto one-dimensional TiO_2 nanocluster/nanorod films for photoactive applications. *Dalton Trans.* **47**, 7237–7244 (2018).

21. Peng, G., Albero, J., Garcia, H. & Shalom, M. A Water-Splitting Carbon Nitride Photoelectrochemical Cell with Efficient Charge Separation and Remarkably Low Onset Potential. *Angew. Chem. Int. Ed.* **57**, 15807–15811 (2018).

22. Fang, Y., Li, X. & Wang, X. Phosphorylation of Polymeric Carbon Nitride Photoanodes with Increased Surface Valence Electrons for Solar Water Splitting. *ChemSusChem* **12**, 2605–2608 (2019).

23. Peng, G., Qin, J., Volokh, M. & Shalom, M. Freestanding Hierarchical Carbon Nitride/Carbon-Paper Electrode as a Photoelectrocatalyst for Water Splitting and Dye Degradation. *ACS Appl. Mater. Interfaces* **11**, 29139–29146 (2019).

24. Karimi-Nazarabad, M., Goharshadi, E. K. & Mahdizadeh, S. J. Efficient Photoelectrocatalytic Water Oxidation by Palladium Doped g-C_3N_4 Electrodeposited Thin Film. *J. Phys. Chem. C* **123**, 26106–26115 (2019).

25. Huang, M., Wang, H., Li, W., Zhao, Y. L. & Zhang, R. Q. In situ textured carbon nitride photoanodes with enhanced photoelectrochemical activity by band-gap state modulation. *J. Mater. Chem. A* **8**, 24005–24012 (2020).

26. Xia, J., Karjule, N., Abisdris, L., Volokh, M. & Shalom, M. Controllable Synthesis of Carbon Nitride Films with Type-II Heterojunction for Efficient Photoelectrochemical Cells. *Chem. Mater.* **32**, 5845–5853 (2020).

27. Karjule, N., Barrio, J., Xing, L., Volokh, M. & Shalom, M. Highly Efficient Polymeric Carbon Nitride Photoanode with Excellent Electron Diffusion Length and Hole Extraction Properties. *Nano Lett.* **20**, 4618–4624 (2020).

28. Abisdris, L. *et al.* Electrophoretic deposition of supramolecular complexes for the formation of carbon nitride films. *Sustain. Energy Fuels* **4**, 3879–3883 (2020).

29. Tashakory, A., Karjule, N., Abisdris, L., Volokh, M. & Shalom, M. Mediated Growth of Carbon Nitride Films via Spray-Coated Seeding Layers for Photoelectrochemical Applications. *Adv. Sustain. Syst.* **11**, 202100005 (2021).

30. Karjule, N. *et al.* Carbon Nitride-Based Photoanode with Enhanced Photostability and Water Oxidation Kinetics. *Adv. Funct. Mater.* **31**, 2101724 (2021).

31. Qin, J. *et al.* Direct growth of uniform carbon nitride layers with extended optical absorption towards efficient water-splitting photoanodes. *Nat. Commun.* **11**, 4701 (2020).

32. Fan, X. *et al.* Coordination Chemistry Engineered Polymeric Carbon Nitride Photoanode with Ultralow Onset Potential for Water Splitting. *Angew. Chem. Int. Ed.* **61**, e202204407 (2022).

33. Karjule, N., Abisdris, L., Azoulay, A., Volokh, M. & Shalom, M. Carbon-Doped Porous Polymeric Carbon Nitride with Enhanced Visible Light Photocatalytic and Photoelectrochemical Performance. *Adv. Energy Sustain. Res.* **2200035**, 2200035 (2022).

34. Li, X., Jiawen Wang, J. X. & Yuanxing Fang, Yidong Hou, Xianzhi Fu, Menny Shalom, and X. W. One-Pot Synthesis of CoS_2 Merged in Polymeric Carbon Nitride Films for Photoelectrochemical Water Splitting. *ChemSusChem* **15**, e202200330 (2022).

35. Karjule, N. *et al.* Photoelectrochemical alcohols oxidation over polymeric carbon nitride photoanodes with simultaneous H_2 production. *J. Mater. Chem. A* **10**, 16585–16594 (2022).

36. Gong, R. *et al.* A Triad Photoanode for Visible Light-Driven Water Oxidation via Immobilization of Molecular Polyoxometalate on Polymeric Carbon Nitride. *Adv. Sustain. Syst.* **6**, 34–41 (2022).

37. Mondal, S. *et al.* Developing extended visible light responsive polymeric carbon nitrides for photocatalytic and photoelectrocatalytic. *Mater. Horizons* **10**, 1363–1372 (2023).

38. Shmila, T. *et al.* Boron and Sodium Doping of Polymeric Carbon Nitride Photoanodes for Photoelectrochemical Water Splitting. *Small* **19**, 2303602 (2023).

39. Zhang, J. *et al.* Direct Growth of Polymeric Carbon Nitride Nanosheet Photoanode for Greatly Efficient Photoelectrochemical Water-Splitting. *Small* **19**, 2208049 (2023).

40. Mondal, S. *et al.* NC Meets CN: Porous Photoanodes with Polymeric Carbon Nitride/ZnSe Nanocrystal Heterojunctions for Photoelectrochemical Applications. *ACS Appl. Mater. Interfaces* **16**, 38153−38162 (2024).

41. Garg, D. *et al.* The Design of Supramolecular Assemblies with Metal Salt as Precursors Enables The Growth of Stable Polymeric Carbon Nitride Photoanodes. *Adv. Sustain. Syst.* **8**, 2300447 (2023).

42. Mondal, S. *et al.* Supramolecular interaction of molecular catalyst on a polymeric carbon nitride photoanode enhances photoelectrochemical activity and stability at neutral pH. *Chem. Sci.* **15**, 16546–16553 (2024).

43. Tashakory, A. *et al.* Minute-Scale High-Temperature Synthesis of Polymeric Carbon Nitride Photoanodes. *Small Struct.* **5**, 2400123 (2024).

44. Mondal, S., Mark, G., Tashakory, A., Volokh, M. & Shalom, M. Porous carbon nitride rods as an efficient photoanode for water splitting and benzylamine oxidation. *J. Mater. Chem. A* **12**, 11502–11510 (2024).

45. Jiang, L. *et al.* What Limits the Stability Performance of Polymeric Carbon Nitride Photoanode for Photoelectrochemical Water Splitting? *Small* **20**, 2403636 (2024).

46. Wu, S. *et al.* Conducting oxide surface engineering enables the growth of a low-defect carbon nitride film for unbiased photoelectrochemical water splitting. *Inorg. Chem. Front.* **12**, 3620–3628 (2025).

47. Rahaman, M. *et al.* Solar-Driven Paired CO_2 Reduction − Alcohol Oxidation Using Semiartificial Suspension, Photocatalyst Sheet, and Photoelectrochemical Devices. *J. Am. Chem. Soc.* **147**, 8168–8177 (2025).

48. Mondal, S. *et al.* Enhanced activity and stability of polymeric carbon nitride photoanodes by yttrium incorporation. *EES Catal.* **3**, 800–810 (2025).

End of Supporting Information
